# *Kansl1* haploinsufficiency impairs autophagosome-lysosome fusion and links autophagic dysfunction with Koolen-de Vries syndrome in mice

**DOI:** 10.1038/s41467-022-28613-0

**Published:** 2022-02-17

**Authors:** Ting Li, Dingyi Lu, Chengcheng Yao, Tingting Li, Hua Dong, Zhan Li, Guang Xu, Jiayi Chen, Hao Zhang, Xiaoyu Yi, Haizhen Zhu, Guangqin Liu, Kaiqing Wen, Haixin Zhao, Jun Gao, Yakun Zhang, Qiuying Han, Teng Li, Weina Zhang, Jie Zhao, Tao Li, Zhaofang Bai, Moshi Song, Xinhua He, Tao Zhou, Qing Xia, Ailing Li, Xin Pan

**Affiliations:** 1 State Key Laboratory of Proteomics, Institute of Basic Medical Sciences, National Center of Biomedical Analysis, Beijing, China; 2 Nanhu Laboratory, Jiaxing, Zhejiang Province China; 3 State Key Laboratory of Toxicology and Medical Countermeasures, Institute of Pharmacology and Toxicology, Beijing, China; 4 Military Institute of Chinese Materia, the Fifth Medical Centre of Chinese PLA General Hospital, Beijing, China; 5 State Key Laboratory of Membrane Biology, Institute of Zoology, Chinese Academy of Sciences, Beijing, China; 6 School of Basic Medical Sciences, Fudan University, Shanghai, China; 7 State Key Laboratory of Experimental Haematology, the Fifth Medical Center of Chinese PLA General Hospital, Beijing, China

**Keywords:** High-throughput screening, Macroautophagy, Gene expression, Neurodegeneration

## Abstract

Koolen-de Vries syndrome (KdVS) is a rare disorder caused by haploinsufficiency of *KAT8 regulatory NSL complex subunit 1* (*KANSL1*), which is characterized by intellectual disability, heart failure, hypotonia, and congenital malformations. To date, no effective treatment has been found for KdVS, largely due to its unknown pathogenesis. Using siRNA screening, we identified *KANSL1* as an essential gene for autophagy. Mechanistic study shows that KANSL1 modulates autophagosome-lysosome fusion for cargo degradation via transcriptional regulation of autophagosomal gene, *STX17*. *Kansl1*^*+/−*^ mice exhibit impairment in the autophagic clearance of damaged mitochondria and accumulation of reactive oxygen species, thereby resulting in defective neuronal and cardiac functions. Moreover, we discovered that the FDA-approved drug 13-cis retinoic acid can reverse these mitophagic defects and neurobehavioral abnormalities in *Kansl1*^*+/−*^ mice by promoting autophagosome-lysosome fusion. Hence, these findings demonstrate a critical role for KANSL1 in autophagy and indicate a potentially viable therapeutic strategy for KdVS.

## Abstract (web summary)

Here the authors show that the Koolen-de Vries syndrome associated gene KANSL1 modulates autophagosome-lysosome fusion via transcriptional regulation of autophagosomal gene Syntaxin17, and that 13-cis retinoic acid can reverses mitophagic defects and neurobehavioural abnormalities of mice lacking Kansl1.

## Introduction

Autophagy is a programmed self-digestion process wherein double-membrane vesicles termed autophagosomes, capture long-lived proteins or organelles such as mitochondria, and fuse with lysosomes to mediate their degradation^1^. This process is critical for maintaining protein and organelle homeostasis in both normal physiological and pathological conditions, particularly within post-mitotic cells that are not protected by the dilutive effects of cell division^2^, such as neurons and cardiomyocytes. It has been reported that genetic ablation of key autophagy genes is sufficient to induce the degeneration of neurons^3–5^, suggesting impaired autophagy may be a driving cause for neurodegenerative diseases. In addition, many proteins that are mutated in neurodegenerative diseases including ALS (Amyotrophic lateral sclerosis), Alzheimer’s disease, and Parkinson’s disease have been implicated in autophagy^6^. In some cases, autophagic failure is considered to be a primary underlying disease pathology^7^. However, many questions and challenges remain unaddressed regarding the underlying molecular mechanisms, as well as the functional and pathophysiological effects of autophagy in diseases at the tissue and organismal levels. Fortunately, expanding knowledge of the complexity of autophagy regulatory machinery and its potential relevance in different diseases can facilitate the development of targeted interventions for these autophagy defect-related diseases.

The *KANSL1* (*KAT8 Regulatory NSL Complex Subunit 1*) gene encodes a nuclear protein that participates in chromatin modification. It has been reported that KANSL1, together with seven other proteins (including KANSL2, KANSL3, PHF20, MCRS1, WDR5, OGT, and HCFC1) form the NSL complex to assist KAT8 (Lysine Acetyltransferase 8; also known as MOF) in the control of pathways that are critical for organismal development and cellular homeostasis^8^. In addition to its function in the nucleus, recent studies suggested that KANSL1 also localizes to microtubules to regulate mitosis^9^ and to mitochondria to affect mtDNA transcription in HeLa cells^10^. However, the full repertoire of KANSL1 biological functions has not yet been elucidated.

Koolen-de Vries syndrome (KdVS) is a rare monogenic disorder characterized by intellectual disability, heart failure, hypotonia, and congenital malformations^11, 12^. To date, no effective treatment has been identified for KdVS, largely due to the unknown pathogenesis of this disease. Although it is well established that the heterozygous mutations in *KANSL1* cause this disease^13, 14^ and a recent study showed that *Kansl1* heterozygous mice indeed display lower levels of recognition memory and brain malformations (thus mimicking the neuronal symptoms of KdVS^15^), it remains unclear how the haploinsufficiency of KANSL1 leads to the multisystem disorders associated with KdVS.

In the present study, we decipher a pathway linking haploinsufficiency of KANSL1, autophagic dysfunction, defective mitochondrial clearance, and impaired neuronal and cardiac function in mice. We also discover that an FDA-approved drug, 13-cis retinoic acid, promotes autophagosome-lysosome fusion and can thus reverse neurodegeneration in *Kansl1* heterozygous mice. These findings expand the pathophysiological relevance of autophagy and also offer a therapeutic strategy for treatment of KdVS.

## Results

### KANSL1 is required for autophagy

**Fig. 1:**
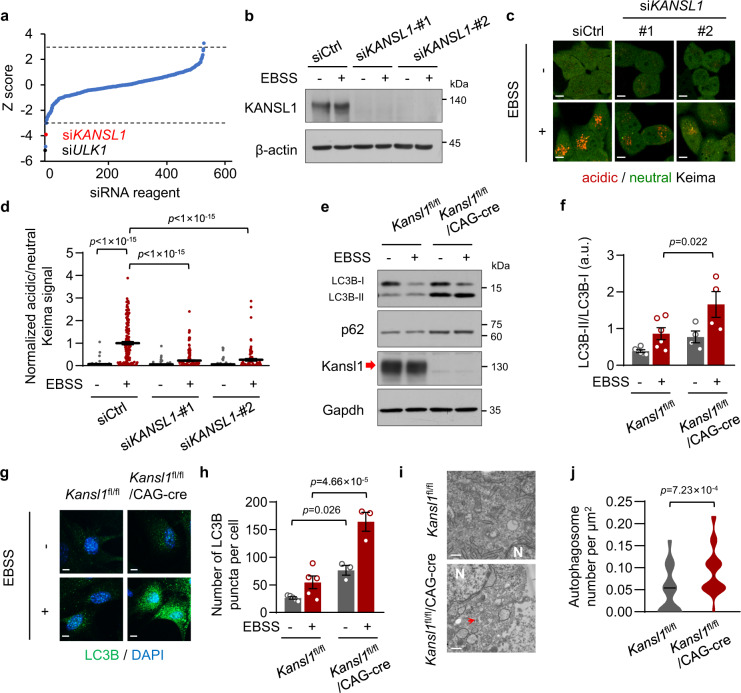
KANSL1 is required for autophagy. **a** Graphical representation of the siRNA screen output. The ‘Z-score’ conveys the distribution of autophagic activity per well across the entire library (see details in “Methods”). Dashed lines are screen-specific cutoffs for active siRNA reagents. Black dots represent positive control siRNAs (si*ULK1*). **b** Western blot analysis of HeLa cells transfected with siRNAs after 12 h of EBSS treatment with the indicated antibodies. **c** Keima imaging in HeLa cells transfected with the indicated siRNAs after 12 h of EBSS treatment. The neutral Keima signal is excited at 458 nm (green) and the acid Keima signal is excited at 561 nm (red). Scale bar, 10 μm. **d** Quantification of Keima signal in (**c**). Values are normalized to the red/green signal in siCtrl with EBSS treatment group. Cells number was obtained from microscopy calculated for 45 to 157 from independent experiments. **e** Primary MEFs (tamoxifen treatment, 1 μM, 48 h) were cultured with EBSS treatment for 6 h. Cell extracts were immunoblotted with the indicated antibodies. The arrow indicated the KANSL1 protein band. **f** Quantitation of protein signal intensities from immunoblots in (**e**) showing the ratio of LC3B-II to LC3B-I (*n* = 6 *Kansl1*^fl/fl^ mice and 4 *Kansl1*^fl/fl^/CAG-cre mice). **g** Representative immunofluorescence images of primary MEFs treated with 1 μM tamoxifen for 48 h and cultured with or without EBSS treatment for 12 h. LC3B was detected as green fluorescent signal and nuclei are labeled with DAPI (blue). Scale bar, 10 μm. **h** Quantification of the number of LC3B puncta per cell in (**g**). *n* = 5 *Kansl1*^fl/fl^ mice and 3 *Kansl1*^fl/fl^/CAG-cre mice. 25–72 cells were collected in each mouse. **i** Electron microscopy images of primary MEFs treated with 1 μM tamoxifen for 48 h. N, nucleus. Arrows indicate autophagosomes. Scale bar, 500 nm. **j** Violin plot showing the number of autophagosomes per μm^2^ in (**i**). *n* = 33 *Kansl1*^fl/fl^ cells and 26 *Kansl1*^fl/fl^/CAG-cre cells. Source data are provided as a Source data file. All data are means ± SEM. **d**, **f** One-way ANOVA with Dunnett’s multiple hoc test; **h** by one-way ANOVA with Tukey’s multiple hoc test; **j** by two-tailed Student’s *t*-tests.

To identify autophagy regulators, we performed an siRNA screen in HeLa cells with a library of 518 different siRNAs^16^ using the pH-sensitive fluorescent protein Keima^17^. The ratio of acid/neutral Keima signal reflects the rate of autophagic degradation of cytoplasmic proteins, thus indicating the autophagic activity (see details in “Methods”). Due to the low basal autophagic activity in cells, we starved HeLa cells with Earle’s Balanced Salt Solution (EBSS) to induce autophagy during screening. We set initial thresholds for putative hits at −3.0 or 3.0 Z-scores to identify positive or negative regulators of starvation-induced autophagy, respectively. We observed a significant decrease in acidic/neutral Keima signal, indicative of an impairment of autophagic degradation of cytoplasmic proteins, following transfection with positive control siRNAs against *ULK1* (Fig. 1a).

In the first round of screening, hits for a total of four positive candidates were below the threshold at −3 Z-score, which suggested that they were potential positive regulators of autophagy. To verify these results, we knocked down these four genes by transfecting Keima-expressing HeLa cells with two siRNAs that target different regions of these genes, which led to a significant decrease in their mRNA levels in cells (Supplementary Fig. 1a). When these cells were cultured in EBSS for 12 h, we observed that starvation induced a marked rise in acidic/neutral Keima fluorescence in all knockdown lines except for the KANSL1-depleted cells (Fig. 1c, d, Supplementary Fig. 1b, c). These results thus suggested that KANSL1 could be essential for autophagic processes.

Next, we utilized murine embryonic fibroblasts (MEFs) derived from wild-type (*Kansl1*^fl/fl^) and tamoxifen-inducible knockout (*Kansl1*^fl/fl^/CAG-cre) embryos to test whether Kansl1 is required for autophagy in primary cells. MAP1LC3B (microtubule-associated protein 1 light chain 3 beta; also known as LC3B) is a widely used biomarker of autophagy^18^. We found that the ratio of LC3B-II/LC3B-I, indicative of autophagosome formation and accumulation, was elevated in *Kansl1*^fl/fl^/CAG-cre MEFs compared with that in WT after treatment with EBSS (Fig. 1e, f). In addition, SQSTM1 (also known as p62), an adapter protein that recruits cargo to autophagic vesicles, was also accumulated to high levels (Fig. 1e). We further observed by immunofluorescence staining that *Kansl1*^fl/fl^/CAG-cre MEFs showed an accumulation of LC3B puncta following starvation (Fig. 1g, h). In line with these results, electron microscopy analyses showed that typical double-membrane autophagosomes accumulated in *Kansl1*^fl/fl^/CAG-cre MEFs (Fig. 1i, j). These results together indicated that KANSL1 deficiency leads to autophagic defects.

### KANSL1 deficiency impairs autophagosome-lysosome fusion

**Fig. 2:**
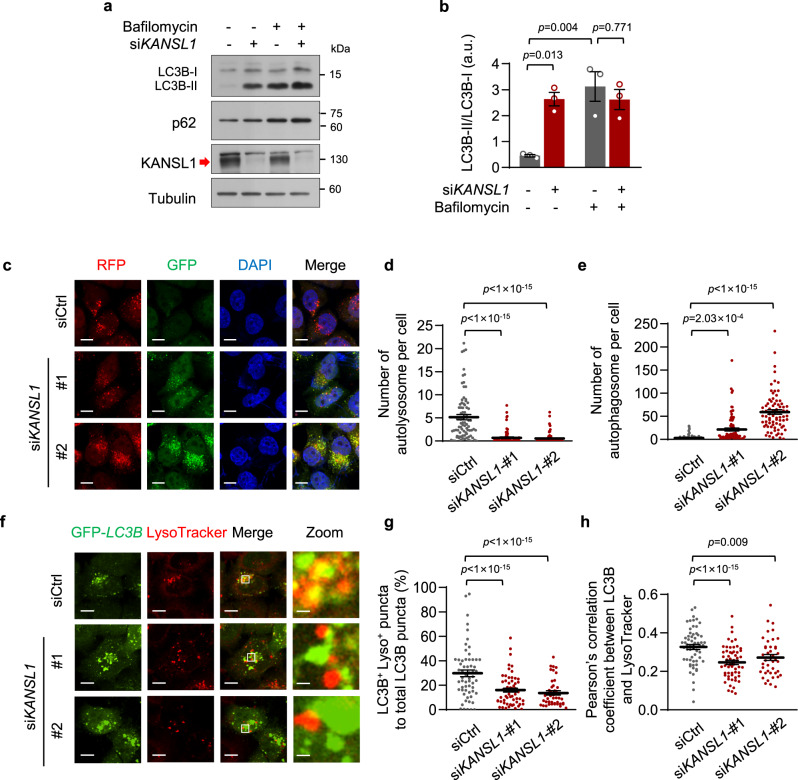
KANSL1 deficiency impairs autophagosome-lysosome fusion. **a** Western blot analysis of HeLa cells transfected with siRNAs with the indicated antibodies. Cells were treated with or without 100 nM bafilomycin A1 for 12 h. The arrow indicated the KANSL1 protein band. **b** Quantitation of protein signal intensities from immunoblots in (**a**) showing the ratio of LC3B-II to LC3B-I (*n* = 3 independent experiments). **c** Representative images of RFP-GFP-LC3B lentivirus-infected HeLa cells transfected with the indicated siRNAs following EBSS (6 h) treatment. Nuclei are labeled with DAPI (blue). Scale bar, 10 μm. **d**, **e** Quantification of the number of autolysosome (**d**) or autophagosome (**e**) per cell in (**c**). *n* = 82, 83, 77 cells. **f** Representative confocal images of colocalization of GFP-*LC3B* puncta and acid lysosome (red, LysoTracker) in HeLa cells transfected with the indicated siRNAs after EBSS treatment for 6 h. **g**, **h** Quantification of the colocalization by calculating the percentage of LC3B^+^ Lyso^+^ puncta to total LC3B puncta (**g**) and Pearson’s correlation coefficient (**h**) in (**f**). *n* = 61, 58, 42 cells. Scale bar, 10 μm and 1 μm in “Zoom”. Source data are provided as a Source data file. All data are means ± SEM. **b** One-way ANOVA with Tukey’s multiple hoc test; **d**, **e**, **g**, **h** by one-way ANOVA with Dunnett’s multiple hoc test.

We next sought to explore the mechanism by which KANSL1 regulates autophagy. In light of our finding that KANSL1 deficiency led to an accumulation of autophagosomes, we speculated that this phenotype could be due to either increased induction of autophagy or blocked degradation of autophagosomes. To explore these two possibilities, we performed autophagic flux assays using the autophagic inhibitor, bafilomycin A1. When autophagic degradation was completely blocked by bafilomycin A1 in HeLa cells, the ratio of LC3B-II/LC3B-I in KANSL1 knockdown cells did not further increase compared to that in control cells (Fig. 2a, b), suggesting that KANSL1 did not affect the induction of autophagic flux. Similar results were also obtained in primary MEFs treated with EBSS and bafilomycin A1 as the ratio of LC3B-II/LC3B-I in Kansl1 knockout cells remains unchanged compared to the wild-type cells (Supplementary Fig. 2). Thus, we hypothesized that KANSL1 deficiency most likely affects autophagosome degradation.

To test this possibility, we visualized autophagosomes and autolysosomes, two key events in autophagy, in HeLa cells stably expressing tandem RFP-GFP-LC3B protein^19^. In this assay, the autophagosomes appeared yellow due to merged red and green fluorescence (RFP^+^GFP^+^ puncta). However, after fusion with acidic lysosomes where the drop in pH quenches the GFP signal, autolysosomes appeared red (RFP^+^GFP^−^ puncta). Using this approach, we found that there was an accumulation of yellow autophagosomes in KANSL1 knockdown cells, whereas the number of red autolysosomes decreased (Fig. 2c–e). This decreased ratio of autolysosomes over total puncta suggested that KANSL1 deficiency disrupts autophagic flux by blocking autophagosome degradation (Supplementary Fig. 3a).

Autophagosome fusion with lysosomes to mediate their degradation is a late-stage event in autophagy. In control HeLa cells, ~30% of autophagosomes colocalized with acid lysosomes after 6 h EBSS treatment, as indicated by LC3B fluorescence labeling and LysoTracker dye staining. By contrast, the colocalization rate of these two markers decreased to ~14% in KANSL1 knockdown HeLa cells (Fig. 2f, g), indicative of decreased autophagosome-lysosome fusion. Similar conclusions were drawn when the colocalization of these two markers was analyzed by calculating Pearson’s correlation coefficient (Fig. 2h). This impairment in autophagosome-lysosome fusion was not likely attributable to lysosomal dysfunction, as KANSL1 depletion did not change the number of acid lysosomes, as visualized by LysoTracker, in cells (Supplementary Fig. 3b, c). These results indicated that KANSL1 likely regulates autophagic flux by contributing to maintaining autophagosome-lysosome fusion.

### KANSL1 regulates *STX17* transcription

**Fig. 3:**
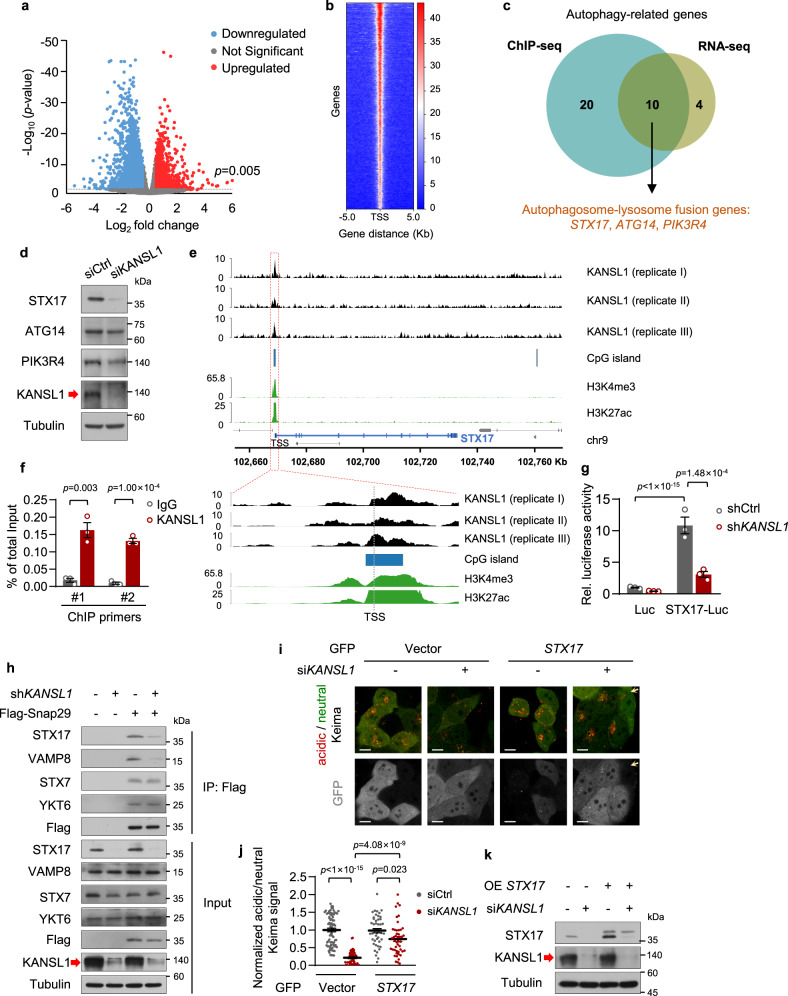
KANSL1 modulates autophagosome-lysosome fusion progress via transcriptional regulation of *STX17*. **a** Volcano plot showing differentially expressed genes (DESeq2 adjusted *p* < 0.005; Log_2_ fold change <−0.5 or >0.5) in *Kansl1*^fl/fl^/CAG-cre compared to *Kansl1*^fl/fl^ MEFs (*n* = 3 mice in each group). **b** A heatmap of input-normalized ChIP peaks for KANSL1. Each peak of gene is represented as a horizontal line. One representative of three independent experiments is shown. **c** Venn diagram showing KANSL1 targeted autophagy-related genes by integrated ChIP-seq and RNA-seq data in (**a**, **b**). **d** Western blot analysis of HeLa cells transfected with siRNAs with the indicated antibodies. The arrow indicated the KANSL1 protein band. **e** pyGenomeTracks representing input-normalized KANSL1 ChIP-seq signal around STX17. ChIP-seq datasets for H3K4me3, H3K27ac, and CpG island are taken from public databases. **f** ChIP-qPCR analysis with KANSL1 antibody showing the enrichment of KANSL1 at the promoter of *STX17* with two different primers in HeLa cells (*n* = 3 technical replicates). The experiment has been replicated for three times with a similar result. **g** Luciferase assay of HeLa cells expressing pGL4.19-*STX17* promoter. *n* = 3 independent experiments. **h** Western blot analysis of co-immunoprecipitation of the Flag-Snap29 in HeLa cells infected with indicated shRNAs lentivirus and cultured in EBSS for 2 h. **i** Keima imaging (neutral signal in green; acid signal in red) in HeLa cells transfected with *KANSL1* siRNA and indicated T2A-GFP-tagged plasmids after 12 h EBSS treatment. Gray signals correspond to cleaved GFP. Arrows indicate *STX17*-T2A-GFP-uninfected cells. Scale bar, 10 μm. **j** Quantification of acidic/neutral Keima signal in (**i**). Cells number was obtained from microscopy calculated for 46 to 74 from independent experiments. Acidic/neutral Keima signal in control cells was normalized to ‘1’. **k** Western blot analysis of HeLa cells transfected with the indicated siRNAs and infected with lentivirus expressing GFP or GFP-*STX17*, where the GFP was cleaved off in cells, as in (**i**). Source data are provided as a Source data file. All data are means ± SEM. **f** Two-tailed Student’s *t*-tests; **g** by one-way ANOVA wi*t*h Dunnett’s multiple hoc test.

Since it has been reported that KANSL1 functions as a transcriptional regulator that targets gene promoters to constitutively activate gene expression^8^, we performed an RNA sequencing assay with WT and tamoxifen-inducible Kansl1 knockout in primary MEFs to investigate the role of KANSL1 in regulating autophagy-related gene expression (Fig. 3a). Among the downregulated genes in Kansl1 knockout MEFs, KEGG analysis showed enrichment for two autophagy-related pathways of the ‘SNARE interactions in vesicular transport’ and the ‘regulation of autophagy’ (*n* = 14 genes) (Supplementary Fig. 4). To investigate the molecular mechanisms of KANSL1 in transcriptional regulation of these genes, we then carried out chromatin immunoprecipitation sequencing (ChIP-seq) in HeLa cells. We detected that KANSL1-binding peaks were enriched within ±5 kb proximal to the transcriptional start sites (TSS) (Fig. 3b). In total, TSS of 3637 overlapping genes were bound by KANSL1 among three independent ChIP-seq analysis. KEGG enrichment analysis of these genes also revealed that the ‘SNARE interactions in vesicular transport’ and the ‘regulation of autophagy’ pathways were enriched, including a total of 30 genes (Supplementary Fig. 5). After cross-referencing RNA-seq data with the ChIP-seq data, we then focused our subsequent functional studies on three of the ten genes that overlapped between analyses: *STX17*, *PIK3R4*, and *ATG14*, which are known to be involved in autophagosome-lysosome fusion^20–22^ (Fig. 3c). Among these three genes, both qPCR and western blot assays revealed that *STX17* was the most significantly decreased in KANSL1 knockdown HeLa cells (Fig. 3d; Supplementary Fig. 6), suggesting that *STX17* could be a downstream target of KANSL1 in regulating autophagy.

To further investigate the mechanism of KANSL1 regulation of *STX17* expression, we analyzed the ChIP-seq data and observed robust KANSL1 binding to a site proximal to the *STX17* promoter, a region where H3K4me3 and H3K27ac histone modification marks, as well as CpG islands, were also enriched based on ENCODE histone mark data taken from the UCSC Genome Browser (Fig. 3e). To confirm these results, we performed a corresponding ChIP-qPCR experiment, which indeed showed that KANSL1 was able to bind to the *STX17* promoter region (Fig. 3f). Moreover, we cloned the KANSL1-binding region in the *STX17* promoter into a luciferase reporter plasmid to verify the effects of KANSL1 on *STX17* transcription. The results showed that KANSL1 deficiency resulted in significant suppression of *STX17* promoter-driven luciferase activity, thus demonstrating that KANSL1 can regulate *STX17* transcription (Fig. 3g).

### KANSL1 regulates autophagy via STX17

The process of autophagosome-lysosome fusion is mediated by two cognate SNARE (soluble N-ethylmaleimide-sensitive factor attachment protein receptor) complexes, STX17-SNAP29-VAMP8^8^ and YKT6-SNAP29-STX7^23^. Given that KANSL1 deficiency downregulated *STX17* transcription levels, we next used co-immunoprecipitation assays to examine whether KANSL1 deficiency also impaired the interactions of autophagosome-lysosome fusion proteins. As expected, the interaction of SNAP29 with STX17 and VAMP8 was inhibited when KANSL1 was depleted in EBSS-treated cells. In contrast, the interaction of SNAP29 with YKT6 and STX7 remained unchanged upon KANSL1 knockdown (Fig. 3h). To further investigate whether KANSL1 regulates autophagy via STX17, we performed rescue experiments in EBSS-treated KANSL1 knockdown HeLa cells with GFP-*STX17* or siRNA-resistant GFP-*KANSL1* plasmids, where the C-terminal tagged GFP was cleaved off in cells. The results showed that that reconstitution of either GFP-*STX17* or GFP-*KANSL1*, could rescue the impaired autophagic activity in KANSL1-deficient HeLa cells as revealed by Keima indicator (Fig. 3i–k; Supplementary Fig. 7a–c). These results suggested that KANSL1 regulates autophagy at least in part via STX17.

It was previously reported that KANSL1 acts as a scaffold protein within the MOF-NSL complex^24^. We therefore investigated whether KANSL1 regulation of *STX17* transcription is dependent on MOF-NSL complex and found that knockdown of KANSL1 in HeLa cells led to a significant decrease in STX17 protein levels, whereas knockdown of MOF per se did not alter STX17 protein levels in cells (Supplementary Fig. 8a). Importantly, in the absence of MOF, KANSL1 depletion still led to a significant decrease in STX17 protein levels (Supplementary Fig. 8a). Similarly, quantification of Keima indicator showed that KANSL1 regulates autophagy independently of MOF in HeLa cells (Supplementary Fig. 8b, c). In addition, we showed that mRNA levels of the other components of the MOF-NSL complex, including *KANSL3*, *WDR5*, or *PHF20* remained unchanged, while *KANSL2* and *MCRS1* mRNA levels showed a marginal increase when KANSL1 was knocked down (Supplementary Fig. 8d). Although these results showed that KANSL1 knockdown resulted in a trend of reduced *MOF* mRNA levels (Supplementary Fig. 8d), MOF protein levels remained unchanged in KANSL1-deficient cells (Supplementary Fig. 8a). Nevertheless, given that knockdown of these genes did not affect autophagic activity in HeLa cells (Supplementary Fig. 8e–g), it was therefore unlikely that KANSL1 regulates autophagy via the MOF-NSL complex.

### *Kansl1*^+/−^ mice exhibit autophagic defects

To evaluate whether Kansl1 is critical for autophagy in vivo, we crossed the *Kansl1*^fl/fl^ mice with EIIA-cre mice to generate whole-body knockout mice. However, when we analyzed more than one hundred live births obtained from parents bearing the *Kansl1*^+/−^ genotype, we did not obtain any viable *Kansl1*^−/−^ progeny (Supplementary Fig. 9a), which suggested that *Kansl1* knockout in mice was embryonically lethal. We noted that the *Kansl1*^+/−^ mice were viable with ~50% reduction in *Kansl1* mRNA levels in various tissues including heart, brain, and skeletal muscle (Supplementary Fig. 9b). Interestingly, *Kansl1*^+/−^ MEFs isolated from *Kansl1*^fl/+^/CAG-cre mice exhibited similar autophagic defects to that observed in *Kansl1*^−/−^ MEFs, as revealed by the accumulation of LC3B puncta (Supplementary Fig. 10a–c). In addition, immunofluorescence staining showed that *Kansl1* deficiency did not further increase autophagosome accumulation in *Stx17* knockdown MEFs (Supplementary Fig. 10a–c), suggesting that Kansl1 haploinsufficiency caused autophagic defects via Stx17.

**Fig. 4:**
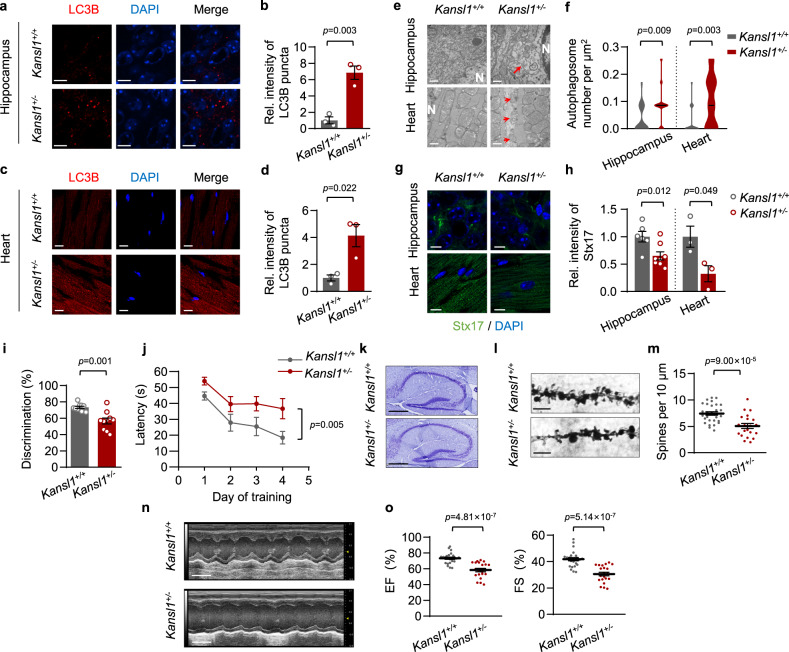
*Kansl1*^+/−^ mice exhibit autophagic defects and impaired neuronal and cardiac functions. **a**, **c** Representative immunofluorescence images of hippocampus CA1 regions (**a**) and hearts (**c**) with LC3B antibody (red). Nuclei are labeled with DAPI (blue). Scale bar, 10 μm. **b**, **d** Quantification of LC3B puncta intensity in (**a**) and (**c**). LC3B puncta intensity in *Kansl1*^+/+^ was normalized to ‘1’ (*n* = 3 mice in each group). **e** Electron microscopy images of hippocampus CA1 regions and hearts. N, nucleus. Arrows indicate autophagosomes. Scale bar, 500 nm. **f** Violin plot showing the number of autophagosomes per μm^2^ in (**e**). *n* = 22, 14, 18, 21 cells from 3 independent experiments. **g** Representative immunofluorescence images of hippocampus CA1 regions and hearts with Stx17 antibody (green). Nuclei are labeled with DAPI (blue). Scale bar, 10 μm. **h** Quantification of Stx17 intensity in (**g**). Stx17 intensity in *Kansl1*^+/+^ was normalized to ‘1’ (*n* = 6, 8, 3, 3 mice from left to right). **i** Novel object recognition test. Discrimination index was calculated as the ratio of time spent exploring the novel object versus both the novel object and the familiar object in the choice trial. *n* = (10 *Kansl1*^+/+^ mice and 10 *Kansl1*^+/−^ mice). **j** Morris water maze test. Mean escape latencies time to find the platform. *n* = 10 *Kansl1*^+/+^ mice and 10 *Kansl1*^+/−^ mice. **k** Representative images of Nissl staining for whole brain of *Kansl1*^+/+^ and *Kansl1*^+/−^ mice. Scale bar, 500 μm. **l** Representative images of dendritic spine of hippocampus CA1 regions visualized with Golgi-cox staining. Scale bar, 5 μm. **m** Quantification of spine density in (**l**). *n* = 29, 21 (from 3 *Kansl1*^+/+^ and 2 *Kansl1*^+/−^ mice). **n** Representative images of echocardiograms on mice. Scale bar, 100 ms. **o** Echocardiographic analyses of (**n**). Left, ejection fraction. Right, fractional shortening. *n* = (24 *Kansl1*^+/+^ mice and 21 *Kansl1*^+/−^ mice). Mice tested above were about 5 months old. Source data are provided as a Source data file. All data are means ± SEM. **b**, **d**, **f**, **h**, **i**, **m**, **o** Two-tailed Student’s *t*-tests; **j** by two-way ANOVA test with Dunnett’s multiple hoc test.

Since KdVS patients have impaired intellectual and cardiac functions, we next evaluated autophagic activities in heart tissue and the hippocampus, a brain region implicated in learning and memory processes in WT and *Kansl1*^+/−^ mice. Indeed, immunofluorescence staining with LC3B antibody, in addition to electron microscopy, revealed an excess of autophagosomes were retained in *Kansl1*^+/−^ hippocampus and heart tissues (Fig. 4a–f). We also demonstrated by immunofluorescence staining that *Kansl1* haploinsufficiency resulted in decreased Stx17 protein levels in these tissues isolated from *Kansl1*^+/−^ mice (Fig. 4g, h). Together, these data indicated that haploinsufficiency of *Kansl1* disrupts autophagic flux in the hippocampus and heart.

### Impaired neuronal and cardiac functions in *Kansl1*^+/−^ mice

Since dysfunctional autophagy frequently leads to neurodegeneration^25–27^ and heart failure^28, 29^, we next investigated the effects of *Kansl1* haploinsufficiency in hippocampal and cardiac functions in mice. We first evaluated the learning and memory ability of *Kansl1*^+/−^ mice using the novel object recognition tests and Morris water maze tests. In the open field, no differences were observed in locomotor activity between *Kansl1* heterozygotes and wild-type littermates (Supplementary Fig. 11a). During the acquisition session, mice of both genotypes spent an equal amount of time exploring the sample object (Supplementary Fig. 11b). After 24 h retention delay, *Kansl1*^+/−^ mice displayed significant memory impairment compared to the wild type during the object recognition task (Fig. 4i). In the Morris water maze task, although *Kansl1*^+/−^ mice could swim as fast as the wild type (Supplementary Fig. 11c), their escape time was significantly longer after 4 days training (Fig. 4j). These results suggested that objection and spatial learning abilities were compromised in *Kansl1*^+/−^ mice. Nissl staining in whole brain sections showed that there was no neuronal loss in *Kansl1*^+/−^ hippocampus (Fig. 4k). However, Golgi-Cox staining revealed a significant reduction in spine density in the hippocampus CA1 region of *Kansl1*^+/−^ mice (Fig. 4l, m), a structural defect that could contribute to hippocampal dysfunction in mice^30^. We also assessed the cardiac functions of *Kansl1*^+/−^ mice by echocardiogram and found that *Kansl1* haploinsufficiency impaired cardiac functions, indicated by decreased ejection fraction and fractional shortening (Fig. 4n, o).

### Impaired mitophagy in *Kansl1*^+/−^ mice

**Fig. 5:**
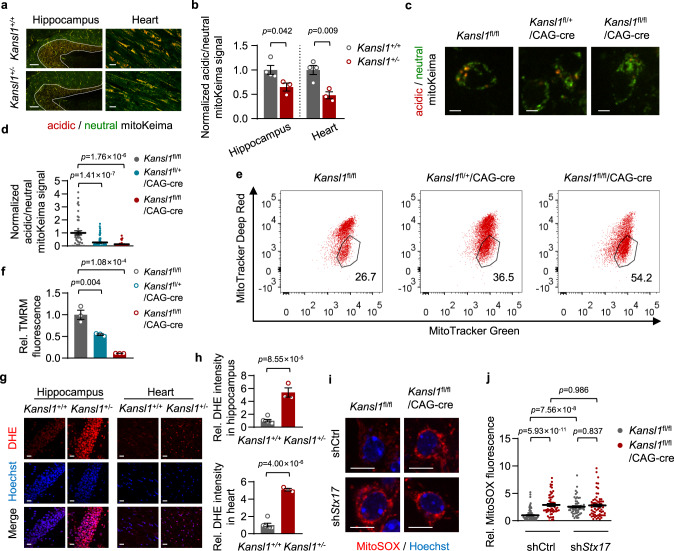
*Kansl1*^+/−^ mice exhibit accumulated damaged mitochondria and elevated ROS levels. **a** mitoKeima signal in the hippocampus and heart of *Kansl1*^+/+^ and *Kansl1*^+/−^ mice. The neutral mitoKeima signal is excited at 458 nm (green) and the acid mitoKeima signal is excited at 561 nm (red). White lines outline hippocampus regions. Scale bar, 120 μm (hippocampus), 25 μm (heart). **b** Quantification of acidic/neutral mitoKeima signal in (**a**). The mitoKeima signal in WT was normalized to ‘1’. *n* = (4 *Kansl1*^+/+^ mice and 3 *Kansl1*^+/−^ mice). **c** mitoKeima imaging (neutral signal in green; acid signal in red) in primary neurons treated with tamoxifen (1 μM, 5 days). Scale bar, 5 μm. **d** Quantification of mitoKeima signal of cells in (**c**). The acidic/neutral mitoKeima signal in WT cells was normalized to ‘1’. *n* = 46, 76, 27 from left to right. **e** Representative FACS plots showing analysis of primary neurons treated with tamoxifen (1 μM, 5 days). Cells were stained with MitoTracker Green and MitoTracker Deep Red at 7 DIV. **f** Quantification of TMRM median intensity by flow cytometry. Primary neurons were treated with tamoxifen (1 μM, 5 days) staining with TMRM. TMRM intensity of WT is normalized to ‘1’ (*n* = 3 mice in each group). **g** Representative fluorescence images of hippocampal CA1 regions and hearts with DHE (red). Nuclei are labeled with Hoechst (blue). Scale bar, 20 μm. **h** Quantification of DHE intensity in (**g**). DHE intensity in *Kansl1*^+/+^ was normalized to ‘1’. *n* = (6 *Kansl1*^+/+^ mice and 3 *Kansl1*^+/−^ mice). **i** Representative confocal images of MitoSOX in primary neurons treated with tamoxifen (1 μM, 5 days). Cells were infected with the indicated lentivirus expressing the shRNAs. Scale bar, 10 μm. **j** Quantification of MitoSOX intensity in (**i**). MitoSOX intensity of WT is normalized to ‘1’. *n* = 102, 55, 56, 66 from left to right. Source data are provided as a Source data file. All data are means ± SEM. **b**, **h** Two-tailed Student’s *t*-tests; **d**, **j** by one-way ANOVA with Tukey’s multiple hoc test; **f** by one-way ANOVA with Dunnett’s multiple hoc test.

Autophagy is important in the maintenance of neurons and cardiac cells, because many cells of the central nervous system and heart are post-mitotic and require precise and exquisitely coordinated quality control systems to eliminate aberrant proteins and damaged organelles. It was previously reported that animals with knockout of core autophagy genes accumulate similar morphologically abnormal mitochondria, especially in mitochondrially-abundant tissues, such as brain and heart^6^. Therefore, we sought to investigate the homeostatic role of Kansl1-mediated autophagy in regulating mitochondrial quality in vivo. To do so, we generated *Kansl1* heterozygous mice expressing mitoKeima, a mitochondrial localization sequence-tagged Keima, in order to observe activity related to selective mitochondrial autophagy, termed mitophagy^31^. As expected, the basal mitophagic levels of *Kansl1*^+/−^ hippocampus and heart declined compared to those in WT (Fig. 5a, b). To confirm the critical role of Kansl1 in mitophagy, we assessed the mitophagic activities in primary neuronal cells. To this end, we first crossed tamoxifen-inducible *Kansl1*^fl/fl^/CAG-cre mice with mitoKeima transgenic mice. The hippocampal cells were dissociated from *Kansl1*^fl/fl^, *Kansl1*^fl/+^/CAG-cre and *Kansl1*^fl/fl^/CAG-cre neonatal mice. These neurons were treated with tamoxifen for 5 days, which resulted in efficient Kansl1 depletion as well as decreased Stx17 protein levels (Supplementary Fig. 12a). As expected, *Kansl1*^fl/+^/CAG-cre and *Kansl1*^fl/fl^/CAG-cre neurons exhibited low mitophagic activity compared with wild type at 7 days in vitro (DIV) (Fig. 5c, d). Notably, infection of lentiviral GFP-tagged *Stx17* into *Kansl1* ^fl/+^/CAG-cre neurons led to partial restoration of mitophagic activity (Supplementary Fig. 12b, c). Although knockdown of Stx17 also inhibited mitophagic activity, our results showed that Kansl1 deficiency did not further downregulate mitophagic activity in the absence of Stx17 (Supplementary Fig. 12d–f), suggesting that Kansl1 regulates mitophagy via Stx17 in neurons.

### Damaged mitochondria accumulate in *Kansl1*-deficient mice

Consistent with the results obtained in HeLa cells, the loss of Kansl1 also blocked the autophagosome-lysosome fusion process in neurons, indicated by reduced colocalization of GFP-LC3B with LysoTracker Red in Kansl1-deficient neurons (Supplementary Fig. 13a, b). In addition, we also observed an increased number of mitochondria captured in autophagosomes (Supplementary Fig. 13c, d), which suggested that damaged mitochondria accumulated in Kansl1-deficient neurons due to the blockage of autophagosome-lysosome fusion. Moreover, we co-stained *Kansl1*^fl/fl^, *Kansl1*^fl/+^/CAG-cre or *Kansl1*^fl/fl^/CAG-cre neurons with MitoTracker Green and MitoTracker Deep Red to label all mitochondria and high MMP (Mitochondrial Membrane Potential) mitochondria, respectively (Fig. 5e). Flow cytometry analysis showed that there was an increase in the number of total mitochondria (MitoTracker Green-positive) and damaged mitochondria (MitoTracker Green-positive, MitoTracker Deep Red-negative) in Kansl1-deficient neurons compared with the WT (Fig. 5e and Supplementary Fig. 14a). In addition, we observed a significant decrease in MMP by TMRM (Tetramethylrhodamine, methyl ester) staining in Kansl1-deficient neurons using flow cytometry (Fig. 5f), which is consistent with the result of MitoTracker Deep Red staining (Supplementary Fig. 14a). These results collectively demonstrated that damaged mitochondria accumulated, rather than undergoing autophagic degradation, in Kansl1-deficient neurons.

Loss of autophagy-related genes typically leads to the accumulation of damaged mitochondria^32–36^. We found that overexpression *STX17* could partially rescue the reduced MMP in Kansl1-deficient neurons (Supplementary Fig. 15a–c). Except for its function in fusion, STX17 has also been reported to localize in mitochondria to mediate mitochondrial division. However, the *STX17* mutant, *STX17-K254C*, which has abolished mitochondrial localization^37, 38^, could also rescue the decline in MMP in these cells to levels similar to those found in the *STX17-WT* controls (Supplementary Fig. 15a–c). This finding indicated that the role of STX17 in KANSL1 regulation of autophagy is unrelated to STX17 mitochondrial localization. Moreover, Kansl1 deficiency did not further reduce MMP in the absence of Stx17 (Supplementary Fig. 15d, e). These results collectively suggested that the defects in STX17-mediated autophagy mainly contribute to the accumulation of damaged mitochondria observed in Kansl1-deficient neurons. Seahorse assays also showed that Kansl1-deficient neurons exhibited lower oxygen consumption rates (OCR) than WT (Supplementary Fig. 16a, b). This reduction in OCR does not likely result from direct damage to mitochondria due to Kansl1 depletion, as mitochondrial complex formation and mitochondrial transcription remained unchanged in *Kansl1*^fl/+^/CAG-cre or *Kansl1*^fl/fl^/CAG-cre neurons compared with that in WT (Supplementary Fig. 16c, d).

### Increased ROS levels in *Kansl1*-deficient mice

Dysfunctional mitochondria have been identified as a major cause of neurodegeneration. They pose a considerable threat to cells because they elevate cellular ROS (reactive oxygen species) levels^6^. Indeed, we showed using fluorescence staining with DHE (dihydroethidium) probe or 8-OHDG (8-hydroxydeoxyguanosine) antibody that ROS levels were significantly increased in *Kansl1* heterozygous hippocampus and heart compared to WT tissues (Fig. 5g, h; Supplementary Fig. 17). Consistent with these results obtained from tissues, we also confirmed by fluorescence dye staining with MitoSOX that Kansl1 depletion induced mitochondrial ROS levels in primary neurons (Fig. 5i, j). In addition, while Stx17 knockdown alone resulted in increased MitoSOX intensity, deletion of Kansl1 did not further increase mitochondrial ROS levels in Stx17 knockdown neurons (Fig. 5i, j), suggesting that Kansl1 deficiency caused ROS accumulation via Stx17 in neurons. Taken together, these results indicate that defects in mitochondrial autophagy could be the potential cause of *Kansl1*^+/−^ mice phenotypes in nervous system and hearts.

### 13-cis RA rescues mitophagic defects in *Kansl1*^+/−^ mice

**Fig. 6:**
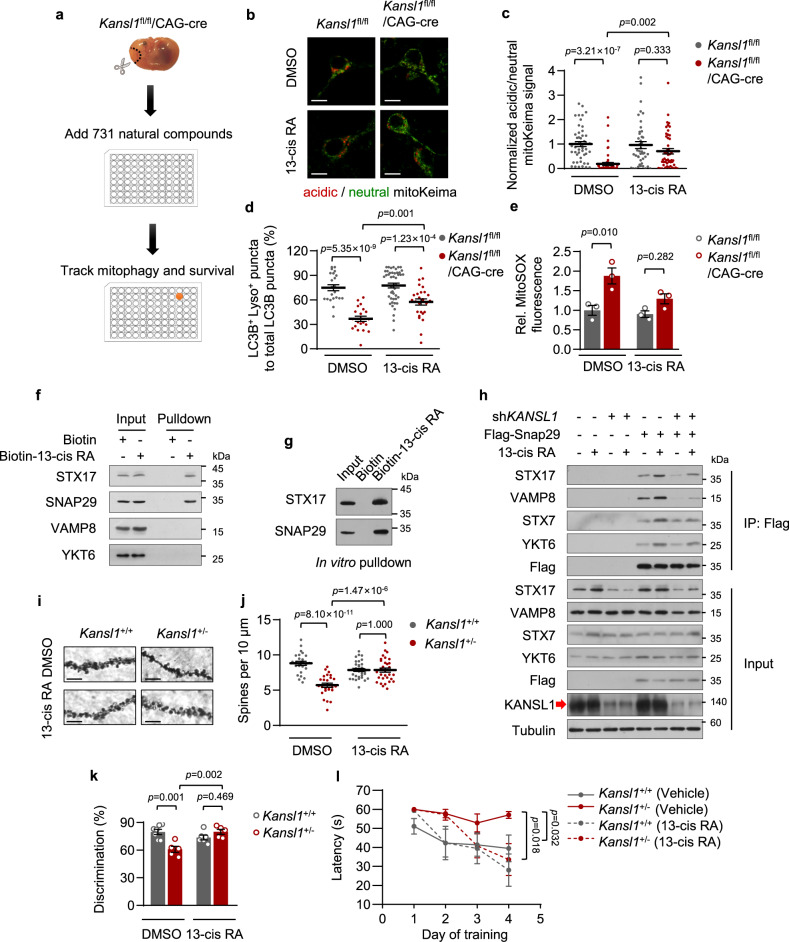
13-cis RA rescues mitophagic defects and reverses neurodegeneration in *Kansl1*^+/−^ mice. **a** Phenotypic screening for small molecules that enhance the mitophagic activity of *Kansl1*^fl/fl^/CAG-cre neurons. **b** mitoKeima imaging in primary neurons treated with tamoxifen (1 μM, 5 days) and13-cis RA (20 μM, 24 h). Scale bar, 10 μm. **c** Quantification of mitoKeima signal in (**b**). The ratio of acidic/neutral mitoKeima signal in control cells was normalized to ‘1’. *n* = 48, 52, 43, 49 from left to right. **d** Quantification of the percentage of LC3B^+^ Lyso^+^ puncta to total LC3B puncta in primary neurons (DMEM, 12 h) treated with 13-cis RA (20 μM, 24 h) (*n* = 22, 21, 46, 27 cells examined in 3 independent experiments from left to right). **e** Quantification of MitoSOX mean intensity by flow cytometry. Primary neurons were treated with tamoxifen (1 μM, 5 days) and cultured with 13-cis RA (2 μM, 7 days). MitoSOX intensity of WT is normalized to ‘1’ (*n* = 3 mice in each group). **f** Western blot analysis of Biotin-13-cis RA pulldown assay. HeLa cells cultured with Biotin-13-cis RA (5 μM, 24 h) were treated with 2 h EBSS. **g** Western blot analysis of in vitro mapping assays among Biotin-13-cis RA and recombinant His-tagged indicated proteins. **h** Western blot analysis of co-precipitation of the Flag-Snap29 with other SNARE proteins in HeLa cells with KANSL1 knockdown. Cells cultured with Biotin-13-cis RA (5 μM, 24 h) were treated with 2 h EBSS. **i** Representative images of dendritic spine of hippocampus CA1 regions visualized with Golgi-cox staining. Scale bar, 5 μm. **j**, Quantification of spine density in (**i**). *n* = 25, 26, 33, 31 (from 2 to 3 mice in each group). **k** Novel object recognition test. For discrimination index, see details in “Methods”. *n* = 7, 5, 6, 5 mice from left to right. **l** Morris water maze test. Mean escape latencies time to find the platform. *n* = 7, 5, 6, 5 mice from left to right. Mice were about 6 months old. Source data are provided as a Source data file. All data are means ± SEM. **c**, **d**, **e**, **j**, **k** One-way ANOVA with Tukey’s multiple hoc test; **l** by two-way ANOVA test with Dunnett’s multiple hoc test.

As there is no effective therapeutic strategy for KdVS, potential drugs are critically required for this disease. We therefore performed a screen to identify small molecules that could rescue the impaired mitophagic activity of Kansl1-deficient neurons (Fig. 6a). We dissociated primary neurons from *Kansl1*^fl/fl^/CAG-cre (KO) mice expressing mitoKeima indicator and then treated them with tamoxifen to induce Kansl1 knockout. At 6 DIV, 731 natural small molecular compound candidates were added to the cells. After 24 h treatment, the mitophagic activities of cells were detected by high-content screening. After excluding those compounds with apparent neuronal toxicity based on cell viability, we identified several compounds that were potentially capable of rescuing the impaired mitophagic activities of Kansl1-deficient neurons from the remaining 559 compounds (Supplementary Fig. 18a). After exclusion of compounds that obviously impacted cell morphology, we selected eight candidates for further verification. Ultimately, 13-cis retinoic acid (13-cis RA) emerged as the most potent candidate among these compounds (Supplementary Fig. 18b, c).

Retinoic acid is the active substance of vitamin A^39^. Vitamin A, also named retinol, can be oxidized to retinoic acid (RA) by a two-step of biochemical reaction^39^. Previous work showed that RA promotes autophagosomal maturation in HeLa cells^40^. To evaluate the effects of 13-cis RA on mitophagy, *Kansl1* WT and KO neurons were cultured in vitamin A-free medium for 24 h and then treated with 13-cis RA for another 24 h. MitoKeima assays showed that the addition of 13-cis RA restored the impaired the mitophagic activity in Kansl1-deleted primary neurons (Fig. 6b, c). Similar results were also obtained when ectopic *Kansl1* was expressed in Kansl1-deficient neurons (Supplementary Fig. 19a, b). Next, to evaluate whether 13-cis RA could promote autophagosome-lysosome fusion in Kansl1-depleted cells, we stained the lysosomes with LysoTracker in primary neurons stably expressing GFP-LC3B. We found that 13-cis RA was able to improve the decreased colocalization of autophagosomes and acid lysosomes in Kansl1 knockout neurons (Fig. 6d). We also noted that supplementation of 13-cis RA decreased mitochondrial ROS accumulation in *Kansl1* knockout primary neurons without affecting cell viability (Fig. 6e; Supplementary Fig. 20).

To further investigate the mechanism by which 13-cis RA rescues mitophagic defects in Kansl1-deficient cells, we first examined the protein levels of Kansl1 and Stx17 in 13-cis RA-treated primary neurons. Western blot analyses showed that 13-cis RA did not affect Kansl1 and Stx17 protein levels (Supplementary Fig. 21). It is known that 13-cis RA targets nuclear receptor RAR and RXR to transcriptionally activate a subset of genes^39, 41^. However, we found that 13-cis RA could still rescue Kansl1 deficiency-mediated autophagic defects when RAR was suppressed by the RAR pan-inhibitor, AGN 194310^42^ (Supplementary Fig. 22a). In addition, docosahexaenoic acid (DHA), an agonist of RXR^43, 44^, did not rescue autophagic defects in Kansl1-deficient cells (Supplementary Fig. 22b). These results suggested that 13-cis RA was not likely to regulate autophagy through its known targets, RAR or RXR.

We then performed pulldown assays followed by mass-spectrometry analysis using Biotin-tagged 13-cis retinoic acid to identify its binding targets in the regulation of autophagy. Mass-spectrometry analysis identified 1176 potential 13-cis retinoic acid-binding proteins, including two key SNARE proteins that mediate autophagosome-lysosome fusion, STX17 and SNAP29 (Supplementary Fig. 23). The binding of 13-cis retinoic acid to STX17 and SNAP29 were further confirmed by both in vivo and in vitro Biotin-pulldown assays (Fig. 6f, g). We also found that 13-cis retinoic acid promotes the interaction of SNAP29 with other SNARE proteins, including STX17, VAMP8, YKT6, and STX7, without increasing their expression levels (Fig. 6h). More importantly, 13-cis retinoic acid partially rescued the impaired interactions of these proteins in Kansl1-depleted cells (Fig. 6h). These results suggested that 13-cis retinoic acid promotes autophagosome-lysosome fusion by facilitating the assembly of SNARE complexes. We also evaluated the potential role of 13-cis RA in general autophagic defects in Atg7 knockdown cells, in which the upstream events for autophagy are inhibited, and found that it produced no protective effects (Supplementary Fig. 24). This finding could be explained by the fact that 13-cis RA promotes autophagy by targeting autophagosome-lysosome fusion but not its upstream events.

### 13-cis RA reverses neurodegeneration in *Kansl1*^+/−^ mice

To determine whether 13-cis RA rescues the KdVS-like phenotype in vivo, we first examined the spine density in the hippocampus CA1 region of *Kansl1*^+/−^ mice. As expected, the reduction in spine density in *Kansl1*^+/−^ mice was rescued by 13-cis RA administration at 0.5 mg/kg/day for 1 month (Fig. 6i, j). Next, we assessed the potential efficacy of 13-cis RA in improving learning and memory impairment in *Kansl1*^+/−^ mice. We found that the impairment of memory in *Kansl1* heterozygous mice was significantly restored by 13-cis RA administration in novel objection recognition tests (Fig. 6k, Supplementary Fig. 25a). In line with this result, 13-cis RA treatment shortened the escape latencies of *Kansl1*^+/−^ mice in Morris water maze tasks (Fig. 6l). This effect was not likely due to changes in the swimming ability of mice, as there was no difference in swimming speed after 13-cis RA treatment (Supplementary Fig. 25b). Thus, these results suggested that 13-cis RA treatment could rescue the autophagic defects in the hippocampus of *Kansl1*^+/−^ mice, thereby promoting their spatial learning and memory ability.

## Discussion

It has long been established that KdVS is caused by the haploinsufficiency of *KANSL1* gene^45^, but little is known about the pathogenesis of this disease. In this study, using a *Kansl1* haploinsufficient mouse model, we provide evidence that the accumulation of damaged mitochondria caused by autophagic dysfunction is a previously unrecognized pathogenesis of KdVS. KdVS is a multisystem disorder in multiple tissues, including brain, heart, and skeletal muscle^14^. It is worth noting that these tissues all harbor abundant mitochondria. The high metabolic rates in these tissues are accompanied by high autophagic activities, which are crucial for the homeostasis of mitochondria and subsequent maintenance of normal tissue functions^46^. Indeed, our study revealed that damaged mitochondria accumulate in the hippocampus and heart tissues of *Kansl1*^+/−^ mice, due to defects in their autophagic activities. As a result, *Kansl1*^+/−^ mice exhibit neurodegeneration and cardiac dysfunction. It is therefore conceivable that Kansl1-mediated autophagy plays an essential role in the clearance of damaged mitochondria in these tissues. Thus, by interrogating a *Kansl1*^+/−^ mouse model, our study links mitophagic defects to KdVS disease. Recently, a study using a human in vitro model for KdVS establishes a link between KANSL1, ROS-mediated autophagy, and synaptic dysfunction. It showed that KANSL1-deficiency leads to increased oxidative stress and autophagic defects in iPSCs and iPSC-derived neurons^47^. In addition, another study using fibroblasts cell lines obtained from KdVS patients also showed a disruption in cellular and metabolic homeostasis associated with this disease^48^. These findings in patient-derived cell lines together with our study on mice are beneficial for a better understanding of the pathogenesis of KdVS. However, studies with tissues from KdVS patients are needed to ultimately validate that these findings represent a bona fide mechanism of pathogenesis.

Lysosomes are degradative organelles, and their fusion with autophagosomes in the autophagic process is strictly regulated. STX17, as an autophagosomal SNARE protein, is required for this fusion step. Although the expression levels of *STX17* are not upregulated during the autophagic process, it cannot be ignored that sufficient expression of STX17 is crucial for the success of this process^8^. In this study, we demonstrate that KANSL1 is essential for autophagosome-lysosome fusion by regulating *STX17* transcription via binding to its promoter. Since it is becoming more important to understand autophagy with a ‘whole-cell’ perspective that encompasses both cytoplasmic and nuclear events^45^, our finding contributes to a better understanding of the nuclear events of autophagy. In addition, although STX17 is important for KANSL1 regulation of the autophagic process, it is worth noting that restoration of STX17 can only partially rescue the autophagic defects in KANSL1-deficient cells. The possibility cannot yet be excluded that KANSL1 regulates autophagy via coordinated modulation of multiple genes. This possibility represents a topic of great interest in future and ongoing studies.

As the pathogenesis of KdVS has remained unknown, no effective treatment strategy is yet available. We discovered that an FDA-approved drug for the treatment of nodulocystic acne^49^, isotretinoin (13-cis retinoic acid), can rescue impaired mitophagic activity in Kansl1-deficient neuronal cells and also reverse the neurobehavioral abnormalities in *Kansl1* heterozygous mice. Therefore, our study provides a potential drug repurposing strategy in treating KdVS. While careful toxicology studies are still needed, there are several pieces of evidence that provide reassurance of the safety of this compound. First, in in vitro studies, 13-cis retinoic acid showed no overt toxicity at any concentration tested, and in fact, it reduced ROS accumulation in Kansl1-deficient cells. Second, in in vivo experiments, the converted human dose of 13-cis retinoic acid used in this study was lower than that of its clinical usage^50^.

It is well-known that drugs with a selective modulation of autophagy/mitophagy, such as Aspirin, Metformin, Digoxin, NAD^+^ precursors, urolithin A, actinonin, and spermidine could be beneficial in the treatment of several diseases through their ability to induce upstream of autophagy/mitophagy^4, 51–54^. However, it is conceivable that upregulating autophagosomal biogenesis is unlikely to be suitable for those genetic diseases caused by impaired autophagosome degradation in the downstream process. Therapies targeting these diseases have been challenging, largely due to the lack of specific agonists for autophagosome-lysosome fusion^55, 56^. In our study, we find that 13-cis retinoic acid promotes the fusion of autophagosomes with lysosomes through direct binding to the core SNARE proteins, STX17 and SNAP29, strengthening their interaction. More importantly, administration of 13-cis retinoic acid could reverse the blockage of downstream autophagic activities, thereby improving neurodegeneration caused by *Kansl1* haploinsufficiency in mice. Thus, these findings highlight that 13-cis retinoic acid could serve as a therapeutic strategy for KdVS and may extend to other diseases caused by downstream blockade of autophagy, which is worthy of future study.

## Methods

### Mouse lines

All animal experiments were performed with the approval of the Institutional Animal Care and Use Committee of Military Medical Sciences (IACUC-DWZX-2020-587). The *Kansl1*^fl/fl^ C57BL/6 mice were generated by Cyagen (Suzhou, China) using the TurboKnockout technique. EIIA-cre (003727), CAG-cre (004682) mice, and mitoKeima transgenic mice (028072) were purchased from the Jackson Laboratory (Bar Harbor, Maine, USA). All mice were backcrossed 10 times onto the B6 background to avoid unpredictable confounders. Mice were maintained in microisolator cages, fed with standard laboratory chow diet and water, and housed in the animal colony. All experiments were conducted with female C57BL/6 mice, 4–5 months old at the start of behavioral training.

### siRNAs and plasmids

*KANSL1*, *NARFL*, *NKAP*, *SPICE1*, *MOF*, *KANSL2*, *KANSL3*, *WDR5*, *MCRS1*, and *PHF20* siRNAs were purchased from Sigma-Aldrich or RIBOBIO and the sequences targeting those siRNA duplexes are provided in Supplementary Table 1. *STX17*, *Stx17*, and *KANSL1* cDNAs as well as RFP-GFP-LC3B lenti-virus were purchased from BioGot (Nanjing, China). We subcloned the cDNAs into pCDH-T2A-copGFP vector to generate GFP tagged *STX17, Stx17*, and siRNA-resistance *KANSL1*, where the C-terminal tagged GFP was cleaved off in cells. *STX17*-K254C mutant was generated by PCR-based site-directed mutagenesis (TransGen Biotech, FM111). The *Snap29* expression construct was generated by PCR amplification from cDNA derived from BMDMs (C57BL/6 mice) and was subcloned into pCDNA3.0 vector to generate Flag-tagged Snap29. The Keima plasmid was a gift from Dr. Toren Finkel.

### Cell lines

HeLa cell line was obtained from American Type Culture Collection (ATCC, CCL-2). Primary MEFs and neurons were isolated and cultured as described^57^. Briefly, primary MEFs were isolated from E12.5 embryos of *Kansl1*^fl/fl^/CAG-cre and *Kansl1*^fl/+^ intercrossed mice. MEFs were cultured in Dulbecco’s modified Eagle’s medium (DMEM) containing 15% fetal bovine serum (FBS). Primary hippocampal or cortical cortex neurons were dissociated from neonates or E16.5 embryos of *Kansl1*^fl/fl^/CAG-cre and *Kansl1*^fl/+^ intercrossed mice. Neurons were grown in Neurobasal medium (Gibco, CA, USA) supplemented with B27 (Gibco, CA, USA), N2 (Gibco, CA, USA), and 100 μg/ml penicillin/streptomycin (Gibco, CA, USA). Cells were treated with 1 μM tamoxifen at 1 day after plating (1 DIV). All cells were cultured under standard conditions (37 °C, 5% CO_2_). For the choice of cell types, we try to use primary MEFs or neurons for most of the experiments. Some of these experiments were also confirmed in HeLa cells to strengthen our conclusion. We use HeLa cells for those mechanistic experiments that involve plasmid transfection, due to the low transfection efficiency of primary cells.

### Drugs

13-cis retinoic acid (TargetMol, T1611) was used for Biotin-coupling, 13-cis retinoic acid (Sigma-Aldrich, R3255) was used for all the other experiments. Spirostan-3-ol (T3S0895), L-Arginine (T3S0364), Ginsenosi-de F3 (T3829), Rosmanol (T7033), Milrinone (T1096), Ampicillin trihydrate (T0814), and Shionone (T5S0691) were purchased from TargetMol. AGN 194310 (hy-16681) and Docosahexaenoic Acid (HY-B2167) were purchased from MCE (MedChemExpress).

### High-content screening

#### siRNAs library

The screening method was described as previous^58^. In brief, pooled siRNAs at a final concentration of 50 nM were used for transfection in HeLa cells stably expressing Keima in 96-well optical plates. 48 h after the siRNA transfection, cells were treated with EBSS for 12 h, and then high-content imaging was acquired using a 20× objective of an Operreta automated microscope (Perkin Elmer, Waltham, MA, USA). Keima localized within the cytoplasm was excited by 410- to 430-nm light and Keima delivered within lysosomes by autophagy was excited by 560- to 580-nm light and both of them were detected by a 590- to 640-nm filter. Images were quantitatively analyzed using Harmony software 4.9. We calculate the ratio of (560–580 nm):(410–430 nm) fluorescence of each single cell. The screen data were normalized using the Z-score method, incorporating the median absolute deviation^59^. The hit cutoff was defined as −3.0 or 3.0.

#### Natural compounds library

Primary cortical cortex cells were dissociated from E16.5 embryos of *Kansl1*^fl/fl^/CAG-cre and seeded in 96-well optical plates. Cells were treated with compounds (66.7 μM final concentration) from natural compounds library purchased by TargetMol at 6 DIV. Keima fluorescence was detected at 7 DIV using a 20× objective of an Operreta automated microscope (Perkin Elmer, Waltham, MA, USA).

### Western blotting

Cells were collected and resuspended in RIPA lysis buffer (50 mM Tris, pH 7.4, 150 mM NaCl, 1% NP-40 [Santa Cruz Biotechnology, CAS 9016–45-9], 0.5% Na-deoxycholate [Sigma-Aldrich, D6750]) containing certain protease inhibitors (0.1 mM phenylmethanesulfonylfluoride [Target Molecule Corporation, T0789], 1× EDTA-free Protease Inhibitor Cocktail [ROCHE, 11873580001]) on ice. Antibodies were used at the following concentrations: rabbit anti-KANSL1 (Abnova, PAB20355; 1:1000) for KANSL1 (Homo sapiens) (Note: this antibody has been purified with the PVDF membrane blotted with Kansl1 knockout MEFs samples before use.), rabbit anti-Kansl1 (AtaGenix, fragments of Kansl1 814-1036 aa were used to immunize rats) for Kansl1 (Mus musculus), rabbit anti-STX17 (Sigma, HPA001204; 1:500), rabbit anti-ATG14 (CST, 5504S, 1:500), rabbit anti-PIK3R4 (CST, 14580S, 1:1000), rabbit anti-VAMP8 (CST, 13060S, 1:500), mouse anti-YKT6 (Santa Cruz Biotechnology, sc-365732, 1:1000), rabbit anti-STX7 (Bethyl, A304-512A, 1:2000), rabbit anti-SNAP29 (Proteintech, 12704-1-AP, 1:1000), rabbit anti-MOF (Bethyl, A300-994A; 1:500), rabbit anti-LC3B (Sigma, l7543; 1:5000), rabbit-p62 (MBL, PM045; 1:5000), rabbit anti-GAPDH (1:5000) prepared in our laboratory and generated by immunizing rabbits with human GAPDH protein, mouse anti-TUBA4A/tubulin (Sigma-Aldrich, T5168; 1:5000), and total OXPHOS Rodent WB Antibody Cocktail (Abcam, ab110413, 1:1000) incubated for 1 h at room temperature or overnight at 4 °C. Membranes were incubated with HRP-conjugated secondary antibodies (Dako Cytomation, P0447 and P0448, 1:5000) for 1 h at room temperature.

### Co-immunoprecipitation

shCtrl and sh*KANSL1* HeLa cells were transfected with the indicated plasmids using TurboFect™ Transfection Reagent (Invitrogen, R0531) and at 24 h post transfection, cells were treated with EBSS for 2 h. For 13-cis retinoic acid rescue experiment, cells were pretreated with 13-cis retinoic acid (5 μM, 24 h) and starved in EBSS for 2 h before harvested. Cells were lysed with M2 buffer. The cell lysates were immunoprecipitated by anti-Flag M2 affinity gel (Sigma-Aldrich, A2220) overnight at 4 °C and washed five times in M2 buffer, and eluted twice using 3× FLAG peptide (Sigma-Aldrich, F4799) at 4 °C for 2 h. Whole-cell lysates and co-precipitation samples were analyzed by western blot.

### Biotin pulldown assay

For in vivo Biotin pulldown assay, HeLa cells were treated with Biotin or Biotin-labeled 13-cis retinoic acid (5 μM, 24 h). Cells were starved for 2 h and harvested with M2 buffer. Streptavidin Agarose beads (Thermo, 20357) were added and incubated overnight at 4 °C. The beads were washed with M2 buffer six times. The indicated proteins were detected by Immunoblotting.

For in vitro Biotin pulldown assay, Biotin or Biotin-labeled 13-cis retinoic acid (0.5 μg) was preincubated with his-tagged STX17 (Proteintech, Ag12378) or SNAP29 (Proteintech, Ag24548) recombinant protein (1.5 μg) for 8 h at 4 °C. Then the mixture was incubated with Streptavidin Agarose beads (Thermo, 20357) overnight at 4 °C. The beads were washed five times with PBS (0.015% Triton X-100) and analyzed by Immunoblotting.

### Mass spectrometric analysis

Protein samples from in vivo Biotin pulldown assay were separated by SDS-PAGE gel then stained with Bio-Safe Coomassie (Bio-Rad). The whole bands were cut down and digested with trypsin. LC-MS/MS analyses were performed on an Easy-nLC 1000 liquid chromatography system (Thermo) coupled to a LTQ-Orbitrap Fusion (Thermo) via a nano-electrospray ion source. Raw file was searched against the human National Center for Biotechnology Information (NCBI) Refseq protein database in Proteome Discoverer 2.1 suited with Mascot software (version 2.3.1, Matrix Science) to achieve a false discovery rate of <1%. The mass tolerance was set to be 20 ppm for precursor, and it was set 50 mmu for the tolerance of productions. Protein identification data (accession numbers, peptides sequence, sequence coverage etc.) are available in Supplementary Data 1.

### Immunofluorescence and confocal microscopy

Fluorescence of Keima was imaged in two channels via two sequential excitations (458 nm, neutral, green; 561 nm, acidic, red) and using a 609- to 735-nm emission range. The ratio of the acidic/neutral Keima signal reflect the underlying level of autophagy. Similarly, the ratio of the acidic/neutral mitoKeima signal reflect the underlying level of mitophagy.

For immunofluorescence analysis, the cells were grown on coverslips and were fixed in 4% paraformaldehyde for 15 min. For LC3B antibody staining, cells were permeabilized with icy methanol and acetone. For Tom20 staining, cells were permeabilized with 0.3% Triton X-100. Newborn goat serum (3%; Thermo Fisher Scientific, 16210072) in PBST (Thermo Fisher Scientific, 28352) was used for blocking. Fixed cells were incubated overnight at 4 °C with rabbit anti-LC3B (Sigma, l7543, 1:400); rabbit anti-Tom20 (Santa Cruz Biotechnology, sc-11415, 1:400). Coverslips were then washed in phosphate-buffered saline (PBS; Gibco, 10010023) and stained for 45 min with Alexa Fluor 488-conjugated secondary antibodies (Invitrogen, A11034; 1:400) or Alexa Fluor 546-conjugated secondary antibodies (Invitrogen, A10040; 1:400). Coverslips were washed again with PBS and mounted with mounting medium containing DAPI (Zsbio Commerce Store, ZLI-9557) or Hoechst 33342 (Invitrogen, H3570).

Dissected organs were fixed in 4% paraformaldehyde prior to optimal cutting temperature embedding. Sections (~8-μm thick) were cut using a cryostat (Thermo Fisher Scientific, CryoStar NX50), mounted on poly-L-lysine-coated microscope slides, and hydrated in PBS. After permeabilized with 0.1% Triton X-100 (Sigma, X100), the sections were incubated in a blocking buffer (5% goat serum, 0.2% Triton X-100 in PBS), and probed with the primary antibodies diluted in the blocking buffer, washed in PBS, followed by the addition of the appropriate secondary antibody diluted in blocking buffer for 30 min. Coverslips were washed again with PBS and mounted with mounting medium containing DAPI (Zsbio Commerce Store, ZLI-9557). Primary antibodies were used at the following concentrations: rabbit anti-STX17 (Sigma, HPA001204; 1:250), rabbit anti-LC3B (Sigma, l7543; 1:400) and mouse-anti-8-Hydroxy-2′-deoxyguanosine (8-OHDG) (Abcam, ab48508; 1:400).

DHE staining was carried out as the manufacturer’s instructions. Tissues were rapidly dissected out, rinsed with PBS, and then rapidly frozen in O.C.T. compound (Tissue-Tek), which were cut in 8 μm sections. Frozen sections were incubated with 50 μM dihydroethidium (DHE, Sigma-Aldrich) at 37 °C for 30 min in a humidified chamber protected from light and washed twice carefully by PBS. Images were taken immediately after mounting them with VectaShield containing Hoechst (Invitrogen, H3570).

TMRM, MitoSOX and LysoTracker staining were carried out as the manufacturer’s instructions. In brief, cells were incubated in a humidified atmosphere with 5% CO_2_ at 37 °C during 15 min to 30 min with TMRM 100 μM (Life Technologies, T668), MitoSOX 100 μM (Life Technologies, M36008), or LysoTracker Red DND-99 50 nM (Life Technologies, L-7528) diluted in warmed culture medium.

Confocal images were obtained using a 60× oil lens objective on an inverted fluorescence microscope (Nikon Eclipse Ti-E, Melville, NY USA) with an UltraView spinning disk confocal scanner unit (Perkin Elmer) for Fig. 1g, Fig. 4a, Fig. 5c, i, Supplementary Fig. 8e, 13a, 17a, 18b, or ZEISS LSM 880 (Zeiss, Germany) for Figs. 1c, 2c, f, 3i, 4c–g, 5a, g, 6b, Supplementary Fig. 1b, 3b, 7a, 8b, 10a, 12, 13c, 15, 19a, 24a. Laser power was set for clearest visualization of the fluorescence signal. Imaging settings were maintained with the same parameters (i.e., pinhole, laser power, and offset gain and detector amplification below pixel saturation) for comparison between different experimental conditions.

### Image analysis

Confocal images were viewed and analyzed using Volocity 6.1.1 software. Background correction was first performed by subtracting the fluorescence intensity of a nearby cell-free region. For images from cells on slides or chambers, quantification analysis was performed by analyzing multiple cells from multiple random visual fields. The fluorescence intensity and colocalization were measured for each cropped cell by Volocity Quantitation. For tissues imaging including LC3B, STX17, DHE as well as 8-OHDG staining, quantification was performed by randomly selecting ~5 visual fields for each mouse. The intensity of fluorescence for each field was measured by Volocity Quantitation. In particular, for mitoKeima image obtained from hippocampus, we first cropped the DG regions of each mouse. A segmentation channel was created by the ratio of 561 nm/458 nm. After subtracting the background of each channel, the ratio value of each picture was calculated as the “acidic/neutral mitoKeima signal”.

### Transmission electron microscopy

MEF cells were washed with PBS and collected by centrifugation at 600 × *g* for 10 min. The cell mass was fixed in 3% glutaraldehyde (Sigma-Aldrich, G5882). Mice were anesthetized and trans-cardialgy perfused according to local institutional guidelines in 3 steps, slightly modified with 3% paraformaldehyde, 3% glutaraldehyde, 0.5% picric acid in 0.1 M sodium phosphate buffer, pH 7.2 for 10 min. Dissected organs were fixed in the same solution for additional 1–2 h at 4 °C, post-fixed in buffered 2% osmium tetroxide (2.5 h, 4 °C), and embedded in Araldite. Ultrathin sections were examined with the H-7650 transmission electron microscope (Hitachi- Science & Technology, Tokyo, Japan).

### Luciferase activity assay

The promoter region of *STX17* (chr9: 102,669,030–102,669,400) was synthesized and inserted into pGL4.19 vector (Promega) to generate the plasmid parental constructs for luciferase activity assay. HeLa cells infected with indicated shRNAs lentivirus were grown in 12-well culture plate to 50% confluence and then co-transfected with either empty pGL4.19-vector or pGL4.19-*STX17* plasmid and SV40 Renilla luciferase plasmids (Promega). Luciferase activity was detected 24 h post transfection using the Dual-Glo Luciferase Assay luciferase kit (Promega) according to the manufacturer’s instructions. Firefly luciferase activity was normalized to Renilla luciferase activity, and data are represented as the mean and standard deviation of three technical replicates. The experiment has been replicated for three times with a similar result.

### RNA isolation and quantitative real-time PCR

RNA was prepared using TRIzol Reagent (Invitrogen, 15596018). Diluted RNA was reverse-transcribed using random primers and M-MLV Reverse Transcriptase according to the manufacturer’s instructions (Promega Corporation, M1701). Quantification of all gene expression was carried out by quantitative real-time PCR using the SYBR Premix Ex Taq kit (TaKaRa, RR820A). GAPDH was used as internal control. The primers were provided in Supplementary Table 1.

### RNA sequencing and analysis

Primary MEFs were isolated from E12.5 embryos of *Kansl1*^fl/fl^/CAG-cre (*n* = 3) and *Kansl1*^fl/fl^ intercrossed mice (*n* = 3). Cells were treated with 1 μM tamoxifen for 48 h. A total amount of 2 μg RNA per sample for each group was used as input material for the RNA sample preparations. Sequencing libraries were generated using NEBNext® Ultra™ RNA Library Prep Kit for Illumina® (#E7530L, NEB, USA) following the manufacturer’s recommendations and index codes were added to attribute sequences to each sample. All samples passed quality control analysis using a Bioanalyzer 2100 (Agilent). High-throughput sequencing was performed using the Illumina NovaSeq 6000 in Annoroad Gene Technology. RNA-seq reads were aligned to the mouse genome (mm10) using STAR, followed by Deseq2 to identify differentially expressed genes. Genes with low expression levels (<1 TPM) in all conditions were filtered out. KEGG pathway enrichment was performed with the downregulated genes with *p* < 0.005 using the edgeR analysis of three RNA-seq biological replicates. RNA-seq data (accession # GSE176424) have been deposited in NCBI’s Gene Expression Omnibus.

### ChIP-sequencing and ChIP-qPCR

ChIP assay was performed according to the manufacturer’s instructions (Cell Signaling, 9003S). Briefly, cells were cross-linked with 1% formaldehyde and stopped by adding glycine. Chromatin was digested by micrococcal nuclease, and the lysate was sonicated with several pulses to break the nuclear membrane. The digested chromatin was incubated with antibody against KANSL1 (Abnova, PAB20355) or rabbit IgG (Cell Signaling, 2729) as a control. Complexes were isolated with protein G beads. Following elution, cross-links were reversed by Proteinase K for 2 h at 65 °C. The released DNA was purified with a column and analyzed by ChIP-sequencing or qPCR, respectively. For ChIP-sequencing, enriched DNA fragments were subjected to library preparation and high-throughput sequencing was performed using the Illumina NovaSeq 6000 in Annoroad Gene Technology. Short reads were mapped to the hg19 reference genome using Bowtie2, and ChIP peaks were called using MACS2, with the input sample as the control. Enrichment heatmaps that surrounded the ChIP peaks were generated using plotHeatmap and signal plotting of individual genes was generated using pyGenomeTracks. For ChIP-qPCR, quantification of binding to the DNA was defined as the percentage of the whole-cell lysate input DNA through SYBR Green (Applied Biosystems) on the Real-Time PCR Detection System (Applied Biosystems) with the specific ChIP primers. Primers used for ChIP-PCR assay are included in Supplementary Table 1. ChIP-seq data (accession # GSE176425) have been deposited in NCBI’s Gene Expression Omnibus.

### Flow cytometric analyses

Primary cerebral cortex neurons from E16.5 embryos at 7 DIV were washed and lifted from the plates using trypsin. Harvested cells were collected in 15 mL conical tubes and centrifuged for 5 min at 800 × *g*. Cell pellet was resuspended in PBS and stained by MitoTracker Green, MitoTracker Deep Red, TMRM, or MitoSOX as the manufacturer’s instructions. In brief, cells were vortexed and incubated at 37 °C for 15 min to 30 min with MitoTracker Green FM 100 nM (Life Technologies, M-7514), MitoTracker Deep Red FM 100 nM (Life Technologies, M22426), TMRM 100 μM (Life Technologies, T668), or MitoSOX 100 μM (Life Technologies, M36008) diluted in PBS. Cells were stained by 7-AAD (BD Pharmingen™, 559925) for 10 min, then they were washed and resuspended in PBS solution for FACS analysis. The gating strategies for different cell populations were shown in Supplementary Fig. 14b. BD FACS Diva 2.0 was used to collect flow cytometry data. FlowJo software (version 10) was used for FACS data analysis.

### Cell viability assay

Primary neuronal cells were seeded into 96-well plates and incubated with 13-cis retinoic acid or CCCP at the indicated concentrations for the indicated time. The cell viability was analyzed with CellTiter-Glo^®^ Luminescent Cell Viability Assay Kit (G7572, Promega) according to the manufacturer’s instruction.

### Mitochondrial oxygen consumption rate measurements

Mitochondrial oxygen consumption rates (OCR) were measured using a XF24 Seahorse Biosciences Extracellular Flux Analyzer. Mouse cortical cortex neurons were plated onto a Seahorse 24-well plate and treated with tamoxifen (1 μM) for 5 days. At 7 DIV, culture media were changed with 175 µl of fresh Seahorse DMEM basal medium 45 min prior to the assay. Seahorse analyzer injection ports contained 2 μM ATP synthase inhibitor oligomycin, or 0.5 μM carbonyl cyanide-p-trifluoromethoxyphenylhydrazone (FCCP), or 1 μM mitochondrial respiratory complex I inhibitor rotenone plus 1 μM complex III inhibitor antimycin A. Basal mitochondrial oxygen consumption rate (OCR) was determined by subtracting the mitochondrial respiration following antimycin A and rotenone treatment from the base line OCR. Maximal mitochondrial OCR was calculated by subtracting the mitochondrial respiration following antimycin A and rotenone treatment from the FCCP-induced OCR. After assays, neurons were immediately stained by DAPI and oxygen consumption rates were normalized to cell number quantified by DAPI staining (OCR, pmol/min/10^5^ cells).

### Nissl staining

1% Pentobarbital Sodium (701O031, Solarbio, China) was used to anesthetize animals (50 mg/kg) by intraperitoneal injection. The brain was removed and kept in 4% paraformaldehyde. After 24 h, the brain samples were taken for paraffin embedding, and cut into 5-μm thick along coronal sections. Slices were put into the oven at 60 °C for 30–40 min, and dehydrated by gradient alcohol, then immersed in Nissl dyeing solution for 20 min. Images were acquired with a 20×/0.75 objective on a Hamamatsu 2.0 HT digital slide scanner; the acquisition software was Nanozoomer 2.0 HT.

### Golgi-cox staining

Golgi staining was performed using the Golgi Staining Kit (FD NeuroTechnologies) according to the manufacturer’s instructions. All procedures were performed under dark conditions. Brains hemispheres used for Golgi-cox staining were immersed in 3 mL mixtures of equal parts of kit solutions A and B and stored at room temperature for 2 weeks. Then, brain tissues were stored in solution C at room temperature for at least 48 h and up to 7 days before sectioning. Solutions A, B, and C were renewed within the first 24 h. Coronal sections of 100 mm were cut with a cryostat (Thermo Fisher Scientific, CryoStar NX50). Each section was mounted with solution C on an adhesive microscope slide precoated with 1% gelatin/0.1% chromalaun on both slides and stained according to the manufacturer’s protocol with the exception that AppliClear (AppliChem) was used instead of xylene. Finally, slices were coverslipped with PermountTM (Thermo Fisher Scientific).

### Novel objection recognition test

The novel object recognition memory is based on the innate preference of rodents to explore novelty. On the first day, mice were habituated to the arena for 30 min at 60 Lux. On the following day, animals were submitted to the first 10-min acquisition trial during which they were individually placed in the presence of two object A. The exploration time of object A (when the animal’s snout was directed toward the object at a distance ≤1 cm) was recorded. A 10-min retention trial (choice trial) was 24 h later. The familiar object (object A) and the novel object (object B) were placed at a distance 10 cm from two open field corners (the distance between the two objects was ~20 cm) and the exploration time of these two objects was recorded. A discrimination index was defined as (tB/ (tA + tB)) × 100. All mice that did not explore the first objects for more than 3 s during the acquisition trial were excluded from the analysis.

### Morris water maze test

The Morris water maze paradigm was used to evaluate spatial learning and memory of mice. The protocol is adapted from the already established protocol^60^. The apparatus consists in a circular pool (150-cm diameter, 60-cm height) filled to a depth of 40 cm with water maintained at 20–22 °C and made opaque using a white aqueous emulsion. An escape platform, made of 6 cm diameter rough plastic, is submerged 1 cm below the water surface. The test began with 4 days of acquisition, 4 trials per day, at 120 Lux. Each trial started with the mice facing the interior wall of the pool and ended when animals climb on the platform or after a maximum searching time of 90 s. The platform was at the same position for all the four trials but starting positions changed randomly between each trial with departures from each cardinal point. Traveled distances to find the platform and swimming speeds were analyzed each day. The distance traveled and duration spent in each quadrant (NW, NE, SW, SE) were recorded. In order to be sure that the mouse used the platform cue, starting position and platform position were changed for each trial.

### Echocardiographic assessment

Transthoracic echocardiographic analysis was performed on 5-month-old mice. Echocardiographic images were obtained using a Visualsonics high-resolution Vevo2100 system (FUJIFILM VisualSonics, Inc, USA). Two-dimensional parasternal long-axis and short-axis views were obtained at the level of the papillary muscle. Diastolic left ventricular posterior wall thickness (LVPW-d) and systolic left ventricular posterior wall thickness (LVPW-s) were measured to calculate the EF and FS. All the measurements were averaged from three consecutive cardiac cycles.

### Statistics and reproducibility

All experiments were repeated independently at least three times with similar results, except the experiments shown in Fig. 1a and Supplementary Figs. 18a and 23, which were performed once. For western blots (Figs. 1b, e; 2a; 3d, h, k; 6f–h; and Supplementary Figs. 2; 7c; 8a; 10c; 12a, d; 15c; 16c; 21) or confocal images (Figs. 1c, g; 2c, f; 3i; 4a, c, g, k, l; 5a, c, g, i; 6b, i; and Supplementary Figs. 1b; 3b; 7a; 8b, e; 10a; 12b, e; 13a, c; 15a, d; 17a; 18b; 19a; 24a), data are representative of three independent experiments. The data are presented as the means ± SEM. Statistical comparisons between only two groups were carried out using two-sided *t*-tests. One-way ANOVA was used for multiple-group comparisons. A two-way analysis of variance (ANOVA) was used for Morris water maze test. Statistical calculations were carried out using GraphPad Prism 8.0.1.

### Reporting summary

Further information on research design is available in the Nature Research Reporting Summary linked to this article.

## Supplementary information

Supplementary Information

Description of Additional Supplementary Files

Supplementary Data 1

Reporting Summary

## Data Availability

Mass spectrometry proteomics data are available in Supplementary Data 1, and have been deposited to the ProteomeXchange Consortium via the PRIDE^61^ partner repository with the dataset identifier PXD029579. High-content screening data are available in Source data. ChIP-seq data and RNA-seq data are deposited in the Gene Expression Omnibus under GSE176425 and GSE176424, respectively. In addition, previously published, publicly available, ChIP-seq datasets for H3K4me3 (#GEO: GSM733682) and H3K27ac (#GEO: GSM733684) have been obtained and used in this manuscript, and CpG island tracking information is obtained from the UCSC genome database table browser. Additional data will be made available upon reasonable request. Source data are provided with this paper.

## Source data

Source Data

## Code availability

All data supporting the findings of this study are available from the corresponding authors upon reasonable request. All analysis codes and reproducible Rmd files related to NGS in this work are available at GitHub (https://github.com/TintingLi/KANSL1_ChIP) [https://github.com/TintingLi/KANSL1_ChIP] and Zenodo (DOI: 10.5281/zenodo.5635447) [10.5281/zenodo.5635447]^62^.
