# Author Correction: *Kansl1* haploinsufficiency impairs autophagosome-lysosome fusion and links autophagic dysfunction with Koolen-de Vries syndrome in mice

**DOI:** 10.1038/s41467-022-29129-3

**Published:** 2022-03-17

**Authors:** Ting Li, Dingyi Lu, Chengcheng Yao, Tingting Li, Hua Dong, Zhan Li, Guang Xu, Jiayi Chen, Hao Zhang, Xiaoyu Yi, Haizhen Zhu, Guangqin Liu, Kaiqing Wen, Haixin Zhao, Jun Gao, Yakun Zhang, Qiuying Han, Teng Li, Weina Zhang, Jie Zhao, Tao Li, Zhaofang Bai, Moshi Song, Xinhua He, Tao Zhou, Qing Xia, Ailing Li, Xin Pan

**Affiliations:** 1 State Key Laboratory of Proteomics, Institute of Basic Medical Sciences, National Center of Biomedical Analysis, Beijing, China; 2 Nanhu Laboratory, Jiaxing, Zhejiang China; 3 State Key Laboratory of Toxicology and Medical Countermeasures, Institute of Pharmacology and Toxicology, Beijing, China; 4 Military Institute of Chinese Materia, the Fifth Medical Centre of Chinese PLA General Hospital, Beijing, China; 5 State Key Laboratory of Membrane Biology, Institute of Zoology, Chinese Academy of Sciences, Beijing, China; 6 School of Basic Medical Sciences, Fudan University, Shanghai, China; 7 State Key Laboratory of Experimental Haematology, the Fifth Medical Center of Chinese PLA General Hospital, Beijing, China

**Keywords:** High-throughput screening, Macroautophagy, Gene expression, Neurodegeneration

Correction to: *Nature Communications* 10.1038/s41467-022-28613-0, published online 17 February 2022.

In the Acknowledgments section, Dr. Hong Zhang (Institute of Biophysics, Chinese Academy of Sciences) was omitted.

The funding from National Key Research and Development Project of China (2021YFA1300200) was omitted.

The original article has been corrected.

