# Supplementary material for: Kansl1 haploinsufficiency impairs autophagosome-lysosome fusion and links autophagic dysfunction with Koolen-de Vries syndrome in mice
Source: Nat Commun. 2022 Feb 17;13:931. doi: 10.1038/s41467-022-28613-0 (PMC8854428; doi:10.1038/s41467-022-28613-0)
Supplement: Supplementary file 1 — Supplementary Information [file 41467_2022_28613_MOESM1_ESM.pdf]

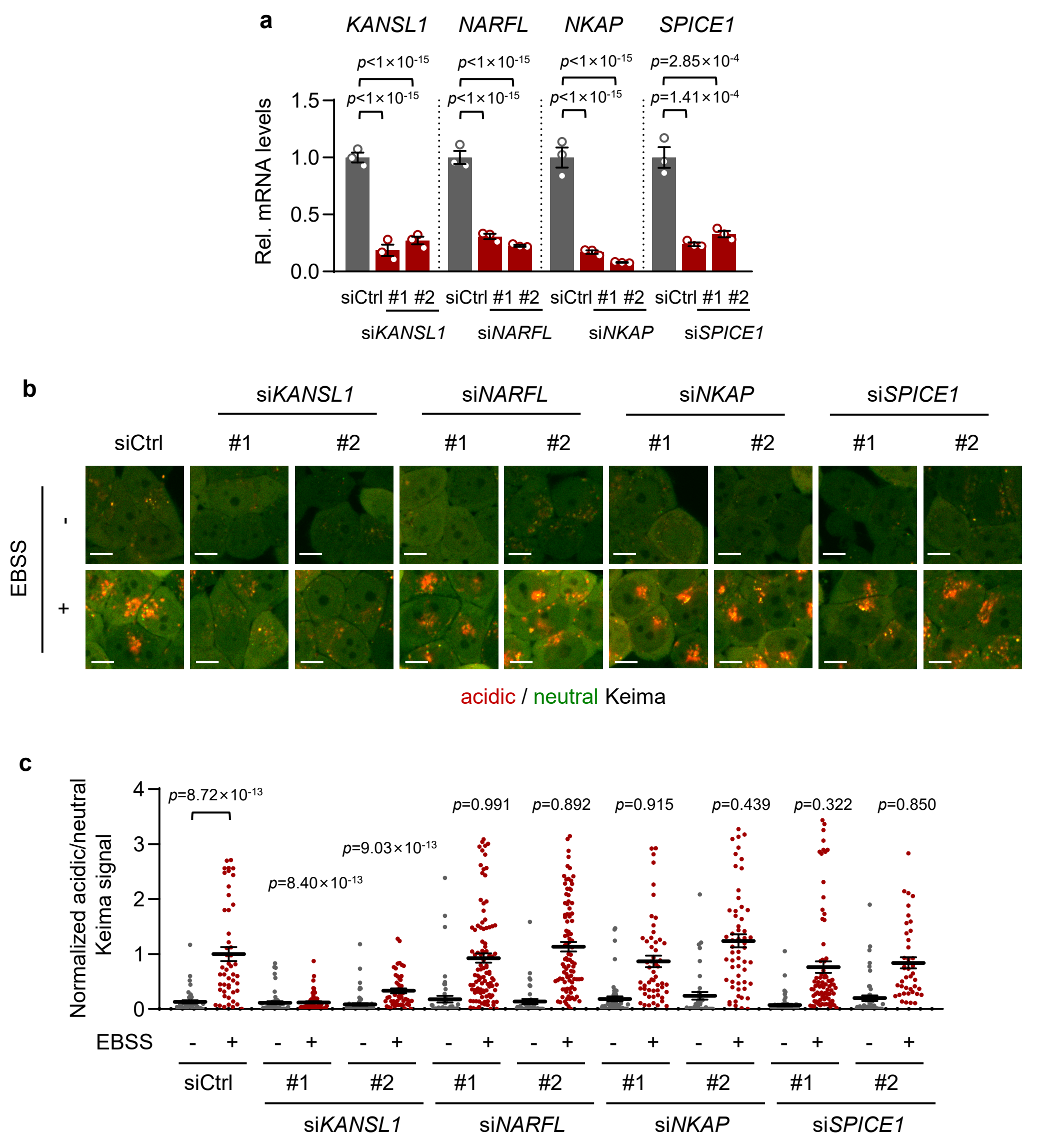

Supplementary Figure 1. Knockdown of KANSL1 inhibits the intensity of acidic/neutral Keima fluorescence. a, Quantitative real-time PCR analysis of mRNA levels for the genes of *KANSL1*, *NARFL*, *NKAP as well as SPICE1* in HeLa cells treated with the indicated siRNAs. (n=3 technical replicates). The experiment has been replicated for three times with a similar result. **b,** Keima imaging in HeLa cells transfected with the indicated siRNAs after 12 h of EBSS treatment. The neutral Keima signal is excited at 458 nm (green) and the acid Keima signal is excited at 561 nm (red). Scale bar, 10 µm. **c,** Quantification of Keima signal in (**b**). Values are normalized to the red/green signal in siCtrl with EBSS treatment group. Cells number was obtained from microscopy calculated for 40 to 101 from independent experiments. All values are compared with the siCtrl group treated with EBSS. Source data are provided as a Source Data file. All data are means ± SEM. (**a**) by one-way ANOVA with Dunnett's multiple hoc test; (**c**) by one-way ANOVA test with Tukey's multiple hoc test.

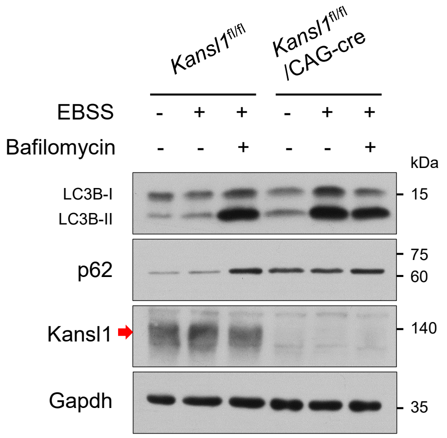

Supplementary Figure 2. Kansl1 deficiency blocks autophagosome degradation. Primary MEFs were treated with 1 µM tamoxifen for 48 h and incubated for 6 h with EBSS in the absence or presence of 20 nM Bafilomycin (24 h). Cell extracts were immunoblotted with the indicated antibodies. The arrow indicated the Kansl1 protein band. Source data are provided as a Source Data file.

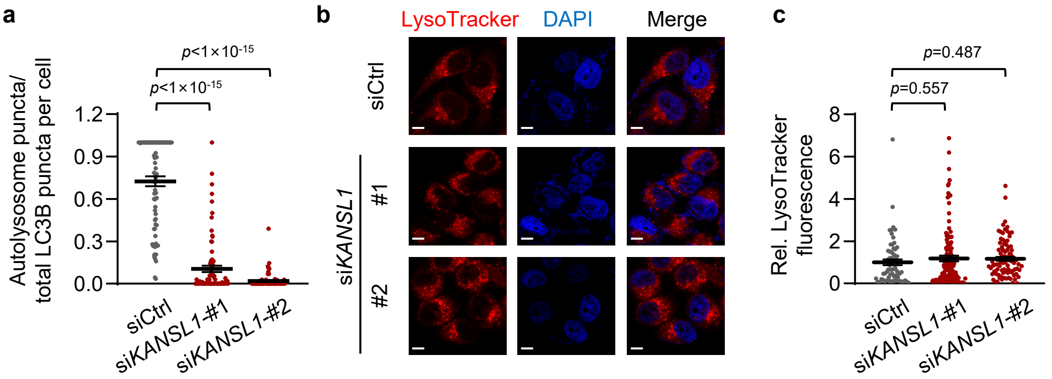

Supplementary Figure 3. KANSL1 deficiency impairs autophagosome-lysosome fusion and does not change the number of acid lysosomes. **a**, Quantification of the ratio of autolysosomes over total LC3B puncta per cell in (Fig.2**c**). n=81,81,77 cells. **b**, Representative confocal images of HeLa cells staining with LysoTracker (red) after EBSS treatment for 6 h. Nuclei are labelled with DAPI (blue). Scale bar, 10 μm. **c**, Quantification of LysoTracker fluorescence intensity of cells in (**b**). LysoTracker intensity in WT cells was normalized to '1'. n=65,118,92 from left to right. Source data are provided as a Source Data file. All data are means ± SEM. (**a**,**c**) by one-way ANOVA with Dunnett's multiple hoc test.

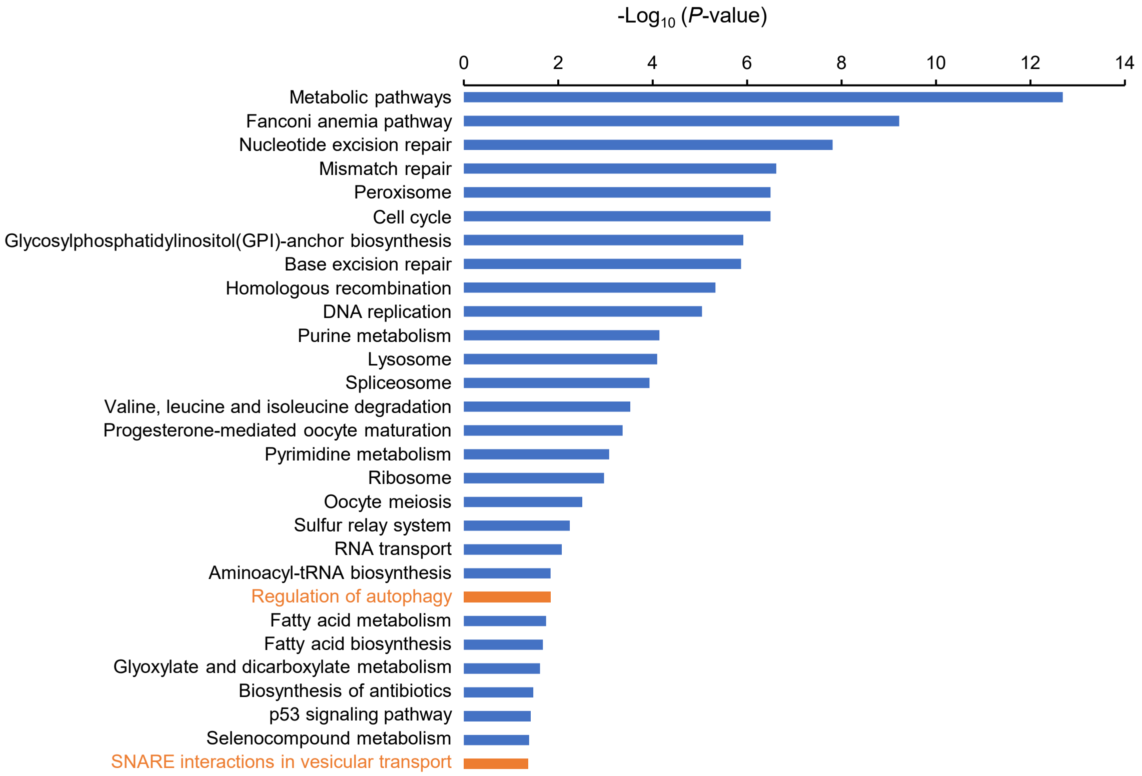

Supplementary Figure 4. KEGG pathway enrichment analysis of downregulated genes in Kansl1 deleted MEFs. KEGG analysis of down-regulated genes in Kansl1 deleted MEFs by RNA-seq results. The enriched KEGG pathways with *P*-value <0.05 are shown. The pathways of SNARE interactions in vesicular transport (mmu04130) and Regulation of autophagy (mmu04140) are highlighted in orange. Source data are provided as a Source Data file.

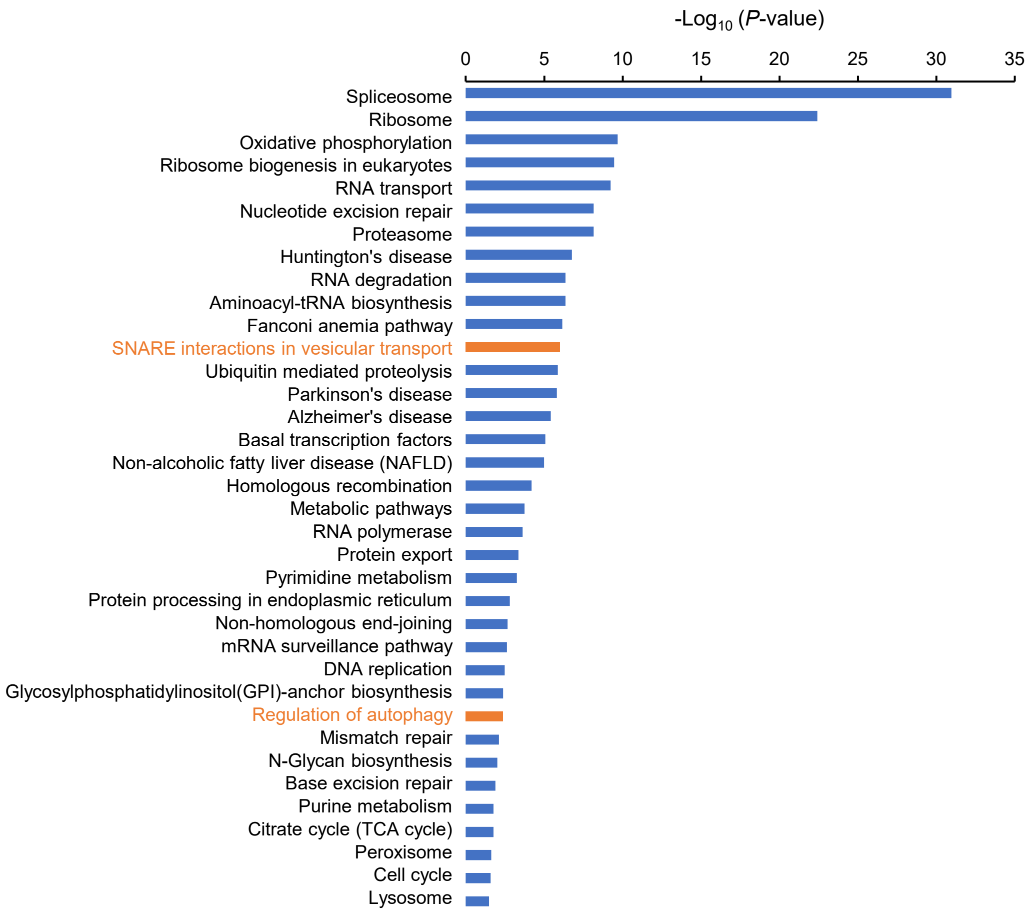

Supplementary Figure 5. KEGG pathway enrichment analysis of direct targets genes of KANSL1. KEGG analysis of genes found in KANSL1 ChIP-seq results. The enriched KEGG pathways with *P*-value <0.05 are shown. The pathways of SNARE interactions in vesicular transport (hsa04130) and Regulation of autophagy (hsa04140) are highlighted in orange. Source data are provided as a Source Data file.

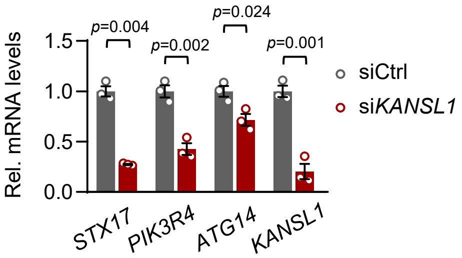

Supplementary Figure 6. KANSL1 deficiency downregulates autophagosome fusion genes mRNA levels. Quantitative real-time PCR analysis of mRNA levels for the indicated genes in (Fig. 3**c**) in HeLa cells treated with siCtrl or si*KANSL1* siRNA (n=3 technical replicates). Source data are provided as a Source Data file. All data are means ± SEM. Data by two-tailed Student's *t*-tests.

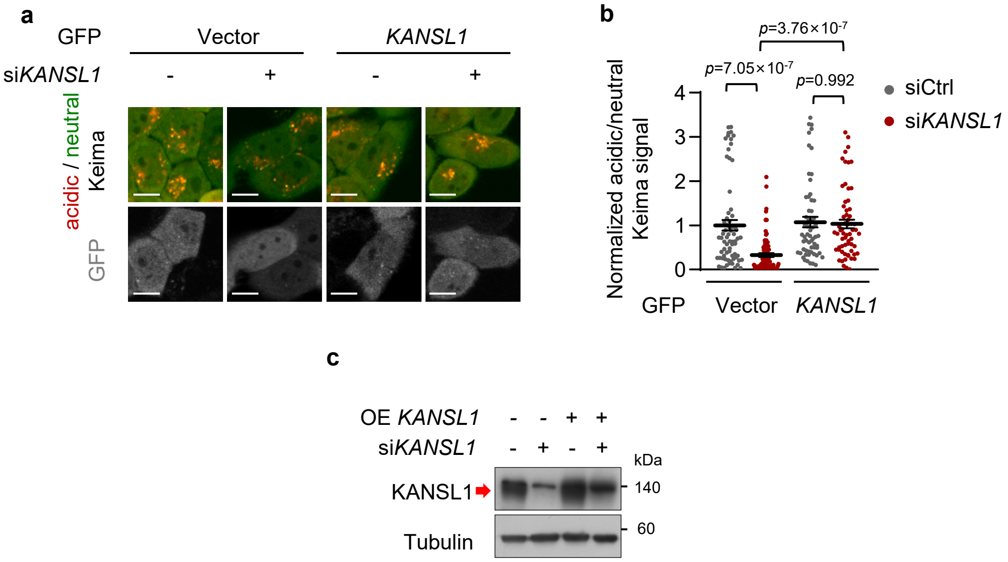

Supplementary Figure 7. Reconstitution of GFP-*KANSL1* is able to restore the impaired autophagic activity in KANSL1-deficient HeLa cells. a, Keima imaging in HeLa cells transfected with *KANSL1* siRNA and infected with lentivirus expressing T2A-GFP-tagged and siRNA-resistant *KANSL1* after 12 h EBSS treatment. Gray signals in the whole cell correspond to cleaved GFP. The neutral Keima signal is excited at 458 nm (green) and the acid Keima signal is excited at 561 nm (red). Scale bar, 10 µm. **b**, Quantification of acidic/neutral Keima signal in (**a**). Acidic/neutral Keima signal in control cells was normalized to '1'. n=70,89,60,64 from left to right. **c**, Western blot analysis of HeLa cells infected with lentivirus expressing T2A-GFP-tagged and siRNA-resistant *KANSL1* plasmid and siRNAs as in (**a**). Western blot analysis was performed for KANSL1 and Tubulin. Source data are provided as a Source Data file. All data are means ± SEM. (**b**) by one-way ANOVA with Tukey's multiple hoc test.

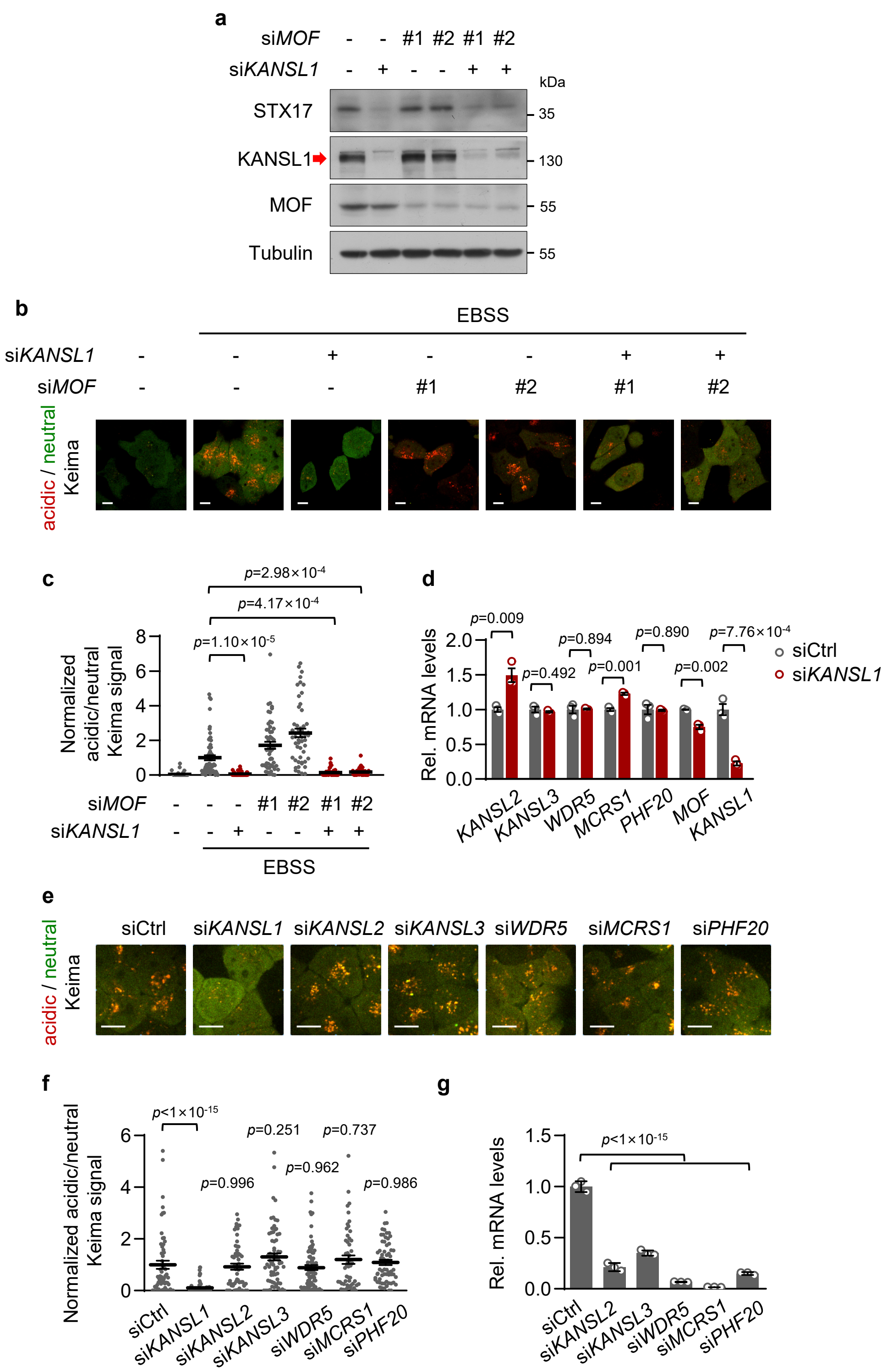

Supplementary Figure 8. KANSL1 regulates autophagy independently of NSL complex. a, Western blot analysis of HeLa cells transfected with siRNAs with the indicated antibodies. **b**, Keima imaging in HeLa cells transfected with the indicated siRNAs after 12 h of EBSS treatment. The neutral Keima signal is excited at 458 nm (green) and the acid Keima signal is excited at 561 nm (red). **c**, Quantification of acidic/neutral Keima signal in (**b**). Cells number was obtained from microscopy calculated for 39 to 64 from independent experiments. Acidic/neutral Keima signal in EBSS-treated control cells was normalized to '1'. **d**, Quantitative real-time PCR analysis of mRNA levels for the indicated genes (*KANSL2*, *KANSL3*, *WDR5*, *MCRS1*, *PHF20, MOF* and *KANSL1*) in HeLa cells treated with siCtrl or si*KANSL1* siRNA. (n=3 technical replicates). **e**, Keima imaging in HeLa cells transfected with the indicated siRNAs after 12 h of EBSS treatment. The neutral Keima signal is excited at 458 nm (green) and the acid Keima signal is excited at 561 nm (red). **f**, Quantification of acidic/neutral Keima signal in (**e**).Cells number was obtained from microscopy calculated for 51 to 80 from independent experiments. Acidic/neutral Keima signal in EBSS-treated control cells was normalized to '1'. **g,** Quantitative real-time PCR analysis of mRNA levels for the indicated genes (*KANSL2*, *KANSL3*, *WDR5*, *MCRS1* and *PHF20*) in HeLa cells. Scale bar, 10 µm. Source data are provided as a Source Data file. All data are means ± SEM; NS, nonsignificant. (**c**) by one-way ANOVA with Tukey's multiple hoc test; (**d**) by two-tailed Student's *t*-tests; (**f**,**g**) by one-way ANOVA with Dunnett's multiple hoc test.

a

**Mating patterns**	***Kansl1***$^{+/-}$ **×** ***Kansl1***$^{+/-}$		
Progeny genotype	*Kansl1*$^{+/+}$ 1	*Kansl1*$^{+/-}$ 2	*Kansl1*$^{-/-}$ 1
The number of each genotype	49	89	0

b

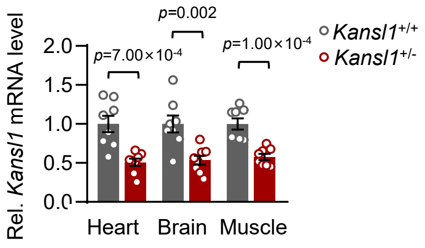

Supplementary Figure 9. *Kansl1* knockout mice are embryonically lethal and *Kansl1* heterozygotes are viable. a, Genotypes of progeny from *Kansl1*$^{+/-}$ × *Kansl1*$^{+/-}$ mattings. No live *Kansl1*$^{-/-}$ embryos were detected postnatally. **b**, Quantitative real-time PCR analysis of *Kansl1* mRNA levels in tissues as indicated (n=8 mice in each group). Source data are provided as a Source Data file. All data are means ± SEM. All by two-tailed Student's *t*-tests.

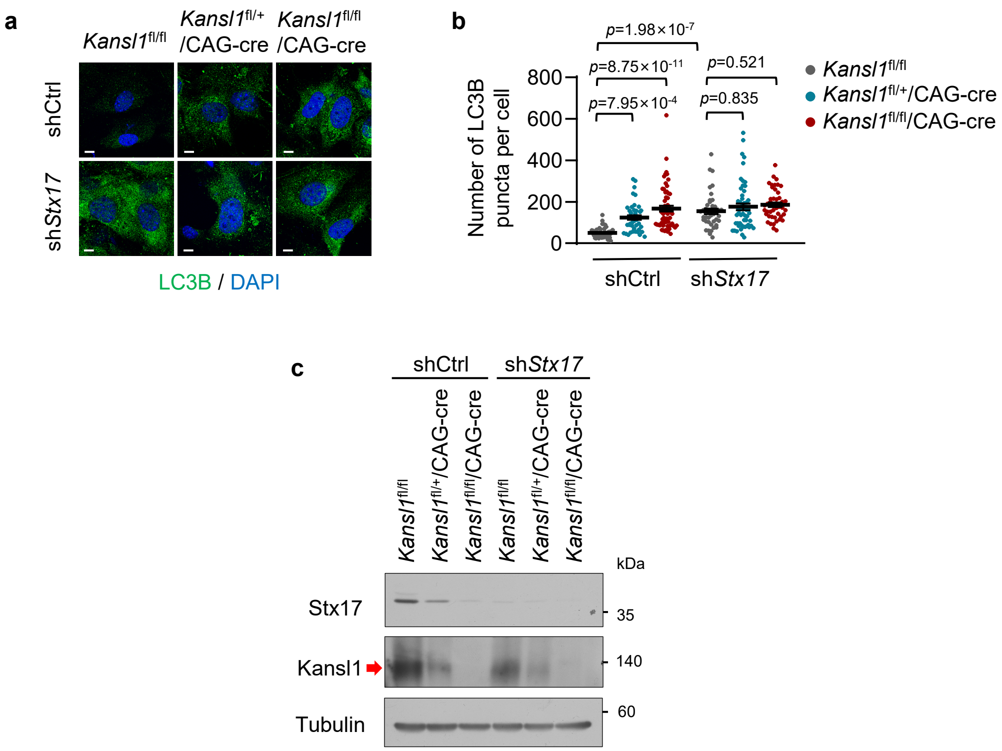

Supplementary Figure 10. *Kansl1* deficiency does not inhibit autophagic activity in the absence of *Stx17*. a, Representative immunofluorescence images of primary MEFs treated with 1 μM tamoxifen for 48 h and cultured with EBSS treatment for 12 h. Cells were infected with indicated lentivirus expressing shRNAs. LC3 was detected as green fluorescent signal and nuclei are labelled with DAPI (blue). Scale bar, 10 μm. **b**, Quantification of the number of LC3B puncta per cell in (**a**). n=51,41,60,42,53,46 cells examined in 3 independent experiments from left to right. All data are means ± SEM. **c**, Western blot analysis of primary MEFs infected with indicated lentivirus expressing shRNAs. MEFs were treated with 1 μM tamoxifen for 48 h. Cell extracts were immunoblotted with the indicated antibodies. Source data are provided as a Source Data file. All by one-way ANOVA with Tukey's multiple hoc test.

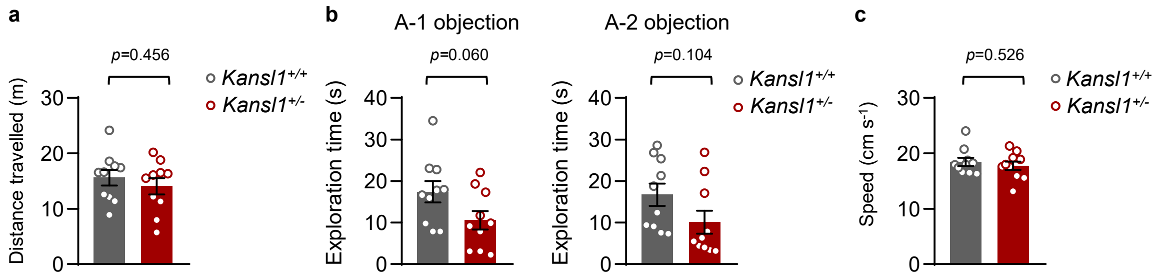

Supplementary Figure 11. *Kansl1* heterozygous mice displayed normal behaviors in tests. a, Open field test. Distance travelled (m) over 10 min of testing. **b**, Object recognition memory of mice was assessed with a retention delay of 24 h. In the first session of test, no difference in object exploration was noticed. **c**, The Morris water maze. No difference of swimming speed was observed in different groups in the Morris water maze. n=10 mice in each group. Source data are provided as a Source Data file. All data are means ± SEM. All by two-tailed Student's *t*-tests.

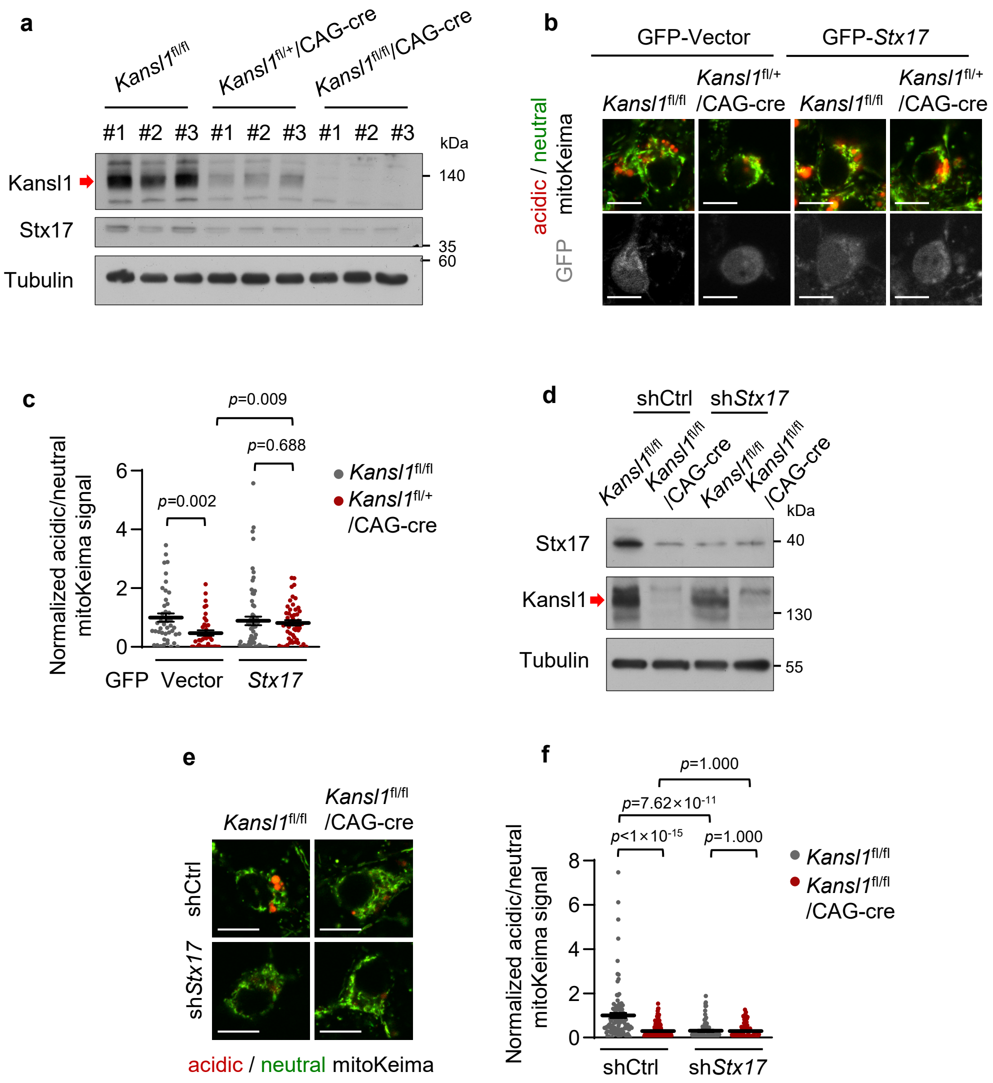

Supplementary Figure 12. Kansl1 regulates mitophagy via Stx17 in primary neurons. a, Primary neurons were treated with 1 µM tamoxifen for 5 days. At 7 DIV, cell extracts were immunoblotted with the indicated antibodies. Each lane represents an individual mouse. The arrow indicated the Kansl1 protein band. **b**, mitoKeima imaging in primary neurons treated with tamoxifen (1 µM, 5 days). Cells were infected with lentivirus expressing T2A-GFP-tagged plasmids. Gray signals in the whole cell correspond to cleaved GFP. The neutral mitoKeima signal is excited at 458 nm (green) and the acid mitoKeima signal is excited at 561 nm (red). **c**, Quantification of mitoKeima signal of cells in (**b**). The acidic/neutral mitoKeima signal in control cells was normalized to ‘1'. n=44,38,63,51 from left to right. Two-sided *t*-tests was used for statistical analysis. **d**, Western blot analysis of primary neurons treated with 1 µM tamoxifen for 5 days. Cells were infected with indicated lentivirus expressing shRNAs. At 7 DIV, cell extracts were immunoblotted with the indicated antibodies. The arrow indicated the Kansl1 protein band. **e**, mitoKeima imaging in primary neurons infected with indicated lentivirus expressing shRNAs treated with 1 µM tamoxifen for 5 days. The neutral mitoKeima signal is excited at 458 nm (green) and the acid mitoKeima signal is excited at 561 nm (red). **f**, Quantification of mitoKeima signal of cells in (**e**). The acidic/neutral mitoKeima signal in control cells was normalized to ‘1'. n=114, 115, 84, 89 from left to right. One-way ANOVA with Tukey's multiple hoc test was used for statistical analysis. Scale bar, 10 µm. Source data are provided as a Source Data file. All data are means ± SEM.

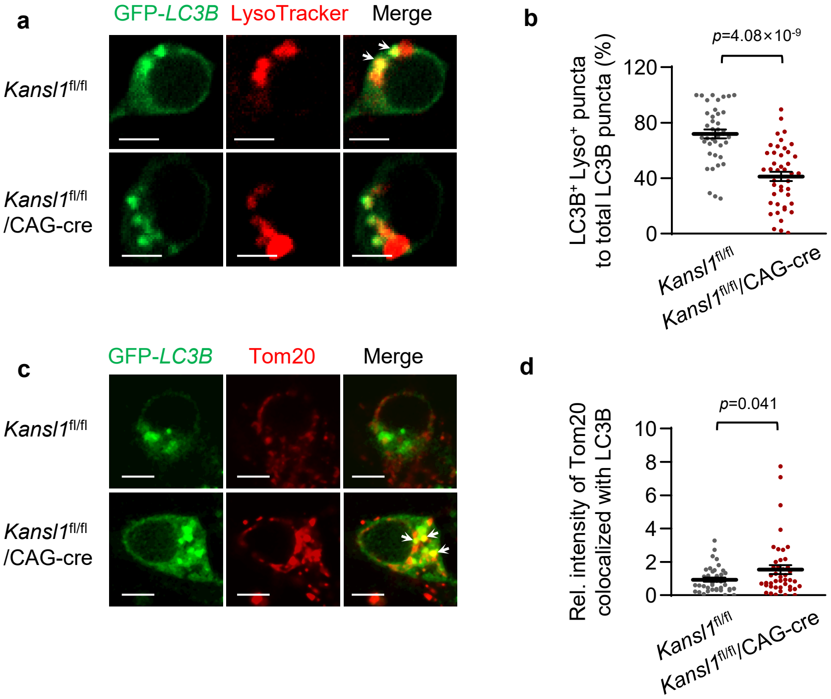

Supplementary Figure 13. Kansl1 deficiency impairs the fusion of autophagosome cargo containing damaged mitochondria with the lysosome. a, Representative images of primary neurons treated with tamoxifen (1 μM, 5 days) and infected with lentivirus expressing GFP-*LC3B* (green). Lysosomes were stained by LysoTracker (red). Arrows indicate LC3B$^+$ Lyso$^+$ puncta. **b,** Quantification of the Colocalization by calculating the percentage of GFP-LC3B puncta containing lysosomes to total GFP-LC3B puncta (**a**). n=40,44 cells. **c,** Representative immunofluorescence images of primary neurons treated with tamoxifen (1 μM, 5 days) and infected with lentivirus expressing GFP-*LC3B* (green). Mitochondria were labelled with Tom20 antibody (red). Arrows indicate Tom20$^+$ LC3B$^+$ puncta. **d,** Quantification of the intensity of Tom20 in autophagosomes. The signal in WT cells was normalized to '1'. n=39,43 cells. Scale bar, 5 μm. Source data are provided as a Source Data file. All data are means ± SEM. All by two-tailed Student's *t*-tests.

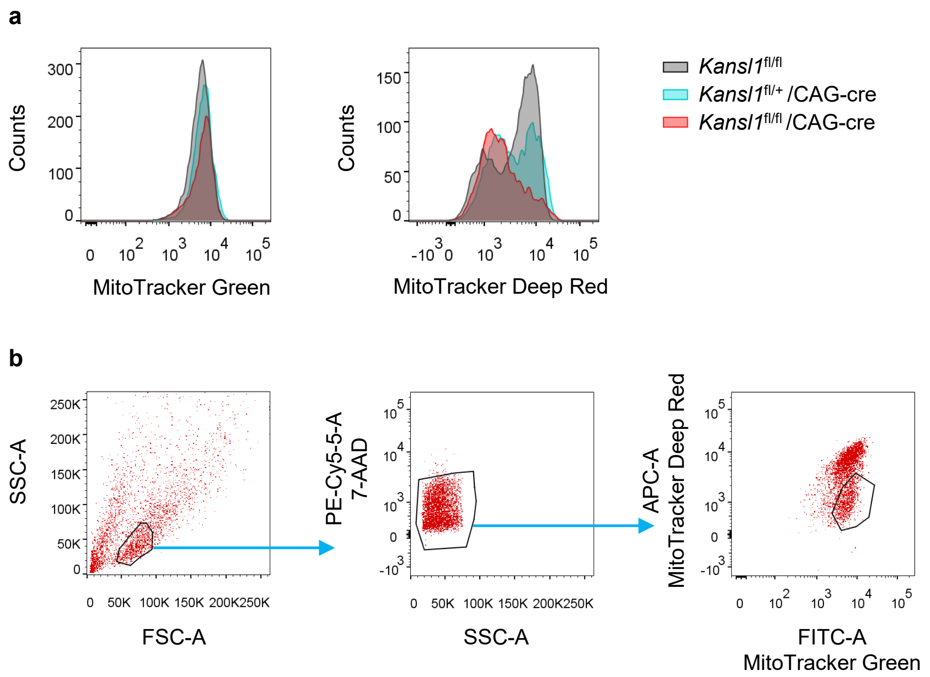

Supplementary Figure 14. Dysfunctional mitochondria accumulate in Kansl1-deficient neurons. **a**, Histograms of FACS analysis of primary neurons treated with tamoxifen (1 μM, 5 days). Cells were stained with MitoTracker Green and MitoTracker Deep Red. **b**, FACS analysis gating strategies of neurons. These gating strategies were also used in main figures from Figure 5 to Figure 6.

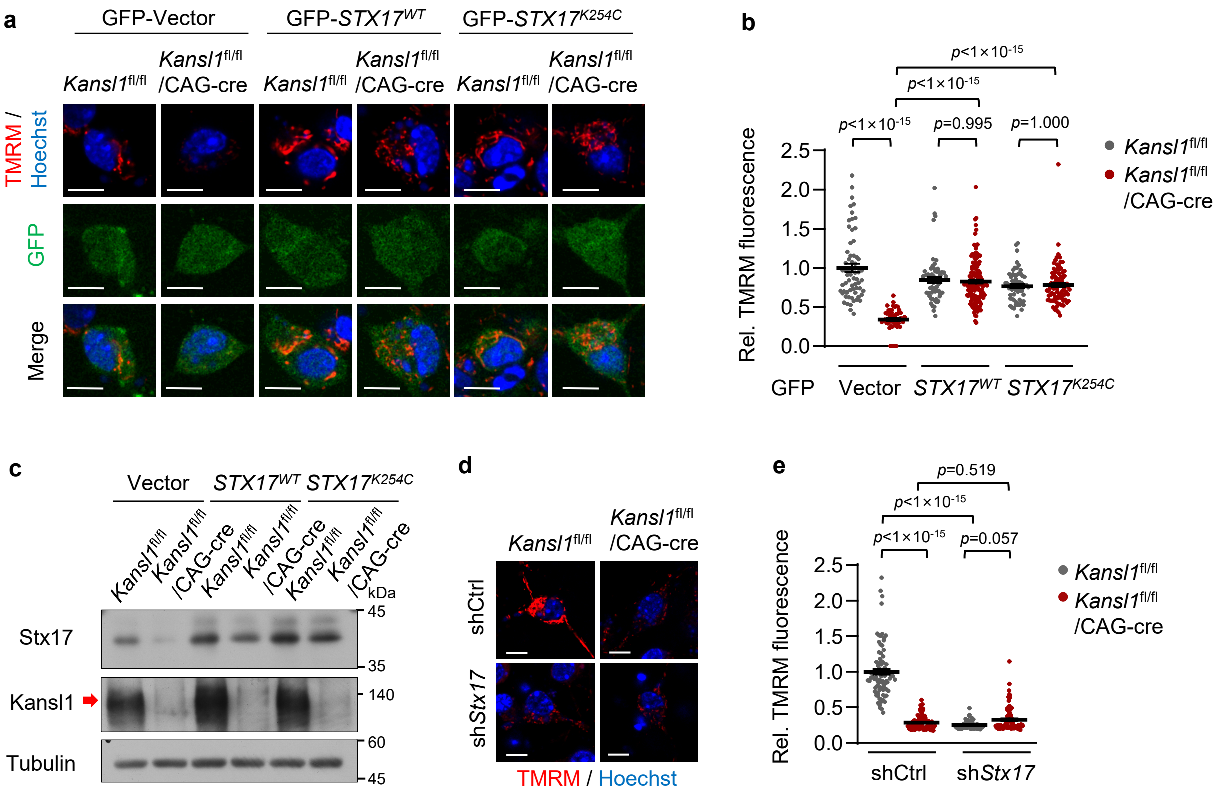

Supplementary Figure 15. The role of Stx17 in Kansl1 regulating autophagy is independent on its function in regulating mitochondrial division **a**, Representative confocal images of TMRM (red) in primary neurons treated with tamoxifen (1 μM, 5 days). Cells were infected with lentivirus expressing GFP-cleaved vector, *STX17*^{WT} or *STX17*^{K254C}. Nuclei were counterstained with DAPI (blue). **b**, Quantification of TMRM fluorescence in (**a**). TMRM intensity of *Kansl1*$^{fl/fl}$ infected with lentivirus expressing GFP-vector is normalized to '1'. n=64, 53, 63, 132, 62, 87 from left to right. **c.** Western blot analysis of neurons in (**a**) with indicated antibodies. The arrow indicated the Kansl1 protein band. **d**, Representative confocal images of TMRM (red) in primary neurons treated with tamoxifen (1 μM, 5 days). Cells were infected with indicated lentivirus expressing shRNAs. Nuclei were counterstained with Hoechst (blue). **e**, Quantification of TMRM fluorescence in (**d**). TMRM fluorescence in control cells was normalized to '1'. n=90, 100, 88, 91 from left to right. Scale bar, 10 μm. Source data are provided as a Source Data file. All data are means ± SEM. All by one-way ANOVA with Tukey's multiple hoc test.

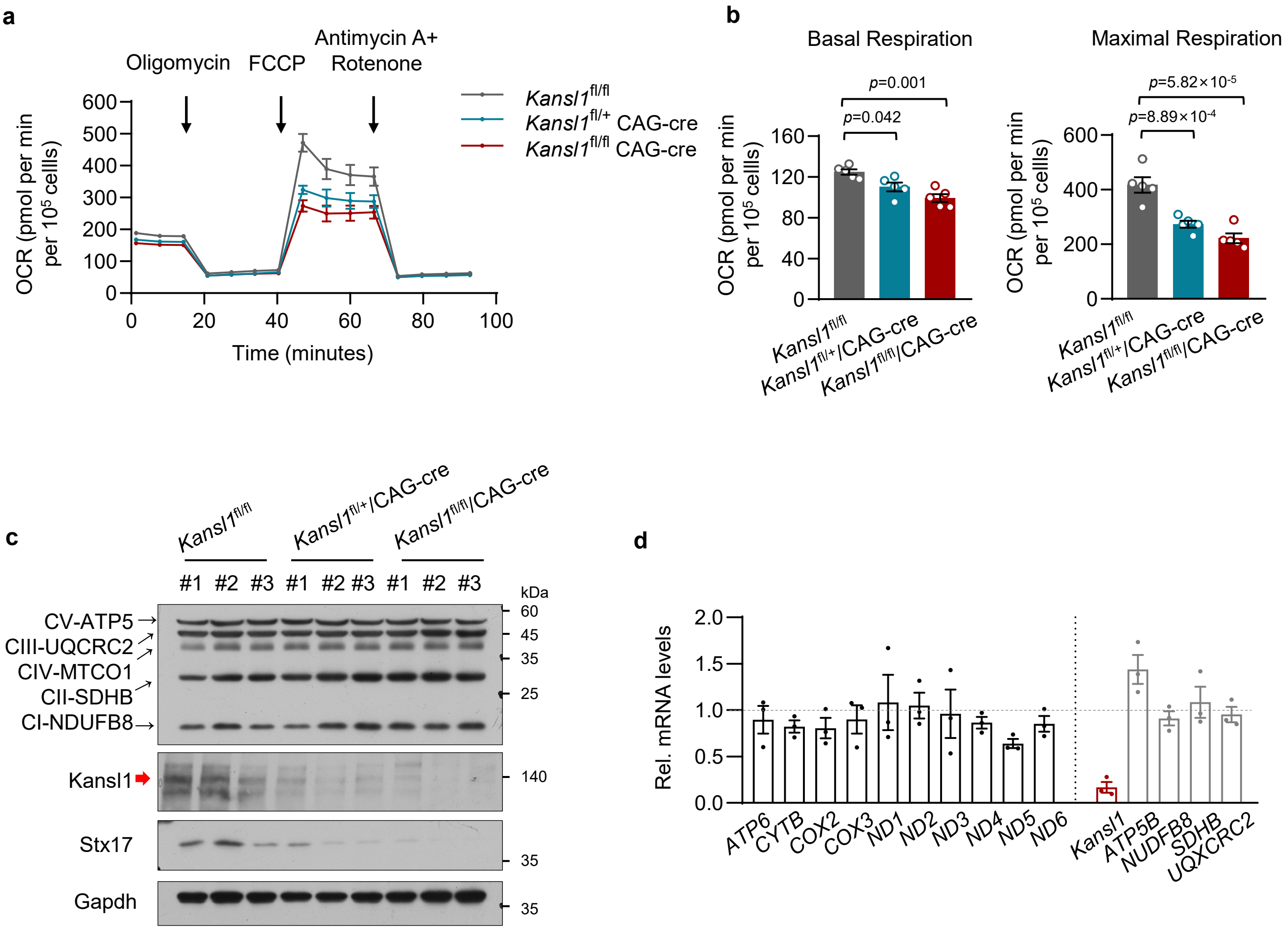

Supplementary Figure 16. Kansl1 deficiency impairs the oxygen consumption rates in neurons, but does not affect mitochondrial complex formations and mitochondrial transcription. a, Seahorse assays showing the oxygen consumption rates (OCR) of primary neurons treated with tamoxifen (1 μM, 5 days) at baseline and in response to oligomycin (2 μM), carbonyl cyanide-p-trifluoromethoxyphenylhydrazone (FCCP) (0.5 μM), and antimycin A (1 μM) plus rotenone (1 μM). n=5 technical replicates. **b**, Statistics of basal and maximal OCR in (**a**). Data are represented as mean ± SEM. **c**, Western blot analysis of OXPHOS in primary neurons treated with tamoxifen (1 μM, 5 days) using an OXPHOS monoclonal antibody cocktail. The arrow indicated the Kansl1 protein band. **d**, Quantification of indicated RNA expression levels in *Kansl1*$^{fl/fl}$/CAG-cre neurons. Each indicated RNA expression level in *Kansl1*$^{fl/fl}$ neurons was normalized to '1'. These RNA levels were normalized to GAPDH. n=3 mice in each group. Source data are provided as a Source Data file. All data are means ± SEM. All by one-way ANOVA with Tukey's multiple hoc test.

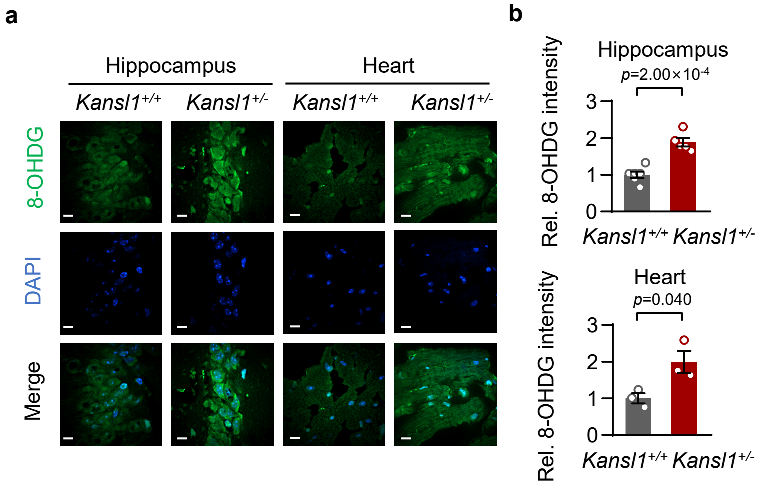

Supplementary Figure 17. Elevated ROS levels in hippocampus and hearts of *Kansl1*$^{+/-}$ mice. a, Representative immunofluorescence images of hippocampal CA1 regions and hearts with 8-OHDG antibody (green). Nuclei are labelled with DAPI (blue). **b**, Quantification of 8-OHDG intensity in (**a**). 8-OHDG intensity in *Kansl1*$^{+/+}$ was normalized to '1'. n= (6 *Kansl1*$^{+/+}$ mice and 5 *Kansl1*$^{+/-}$ mice) for hippocampus and n= (3 *Kansl1*$^{+/+}$ mice and 3 *Kansl1*$^{+/-}$ mice) for heart. Scale bar, 10 µm. Source data are provided as a Source Data file. All data are means ± SEM. All by two-tailed Student's *t*-tests.

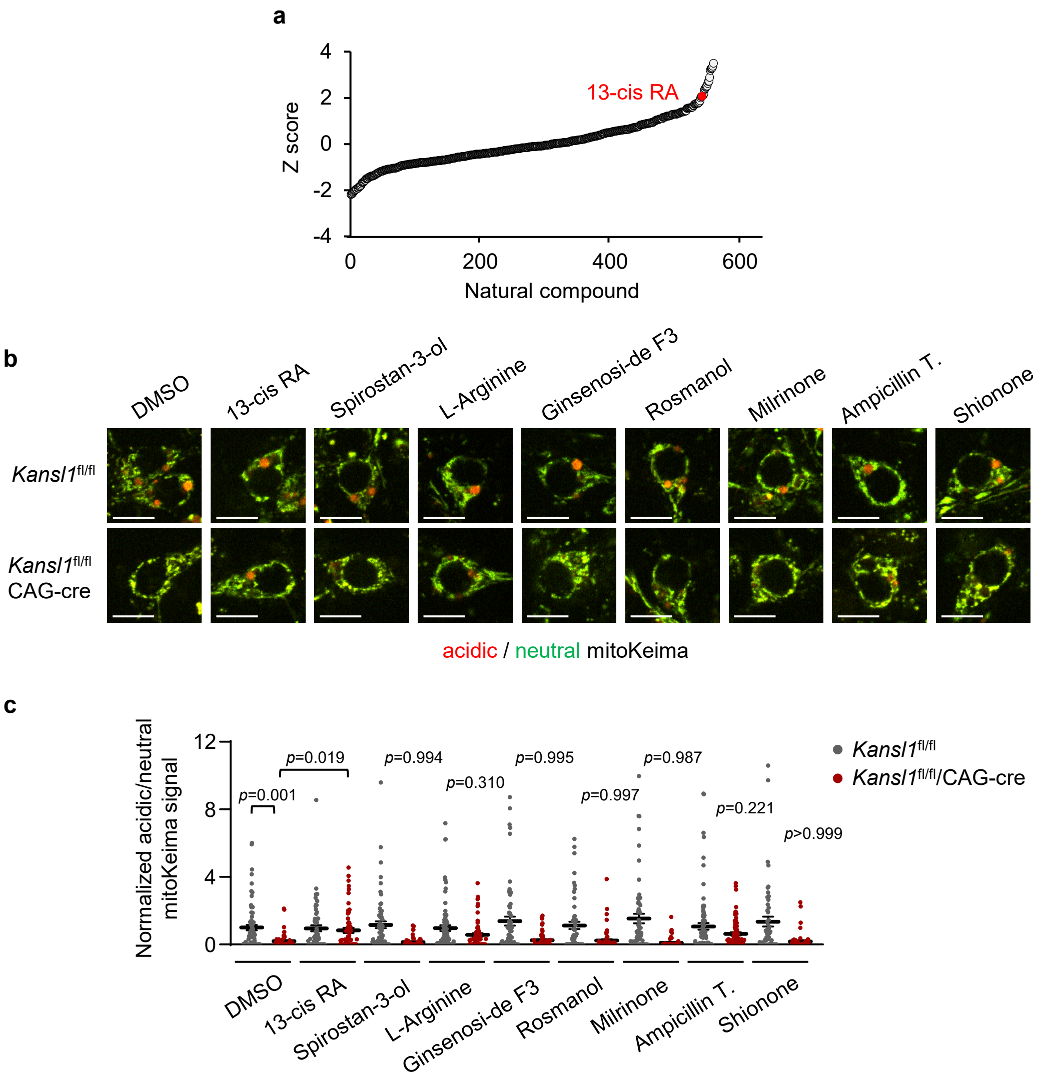

Supplementary Figure 18. 13-cis retinoic acid is identified to rescue impaired mitophagic activity of Kansl1 deficient neurons. a, Graphical representation of the natural products screen output. The 'Z-score' conveys the distribution of mitophagic activity per well across the entire library (see details in Methods). Data ordered from most negative to positive. **b**, mitoKeima imaging in primary neurons treated with tamoxifen (1 μM, 5 days). Cells were treated with the indicated compounds (20 μM, 24 h). The neutral mitoKeima signal is excited at 458 nm (green) and the acid mitoKeima signal is excited at 561 nm (red). Scale bar, 10 μm. **c**, Quantification of mitoKeima signal in (**b**). The acidic/neutral mitoKeima signal in control cells was normalized to '1'. Cells number was obtained from microscopy calculated for 52 to 86 from independent experiments. All values are compared with *Kansl1*$^{fl/fl}$/CAG-cre (DMSO) group. Source data are provided as a Source Data file. All data are means ± SEM; NS, nonsignificant. All by one-way ANOVA with Tukey's multiple hoc test.

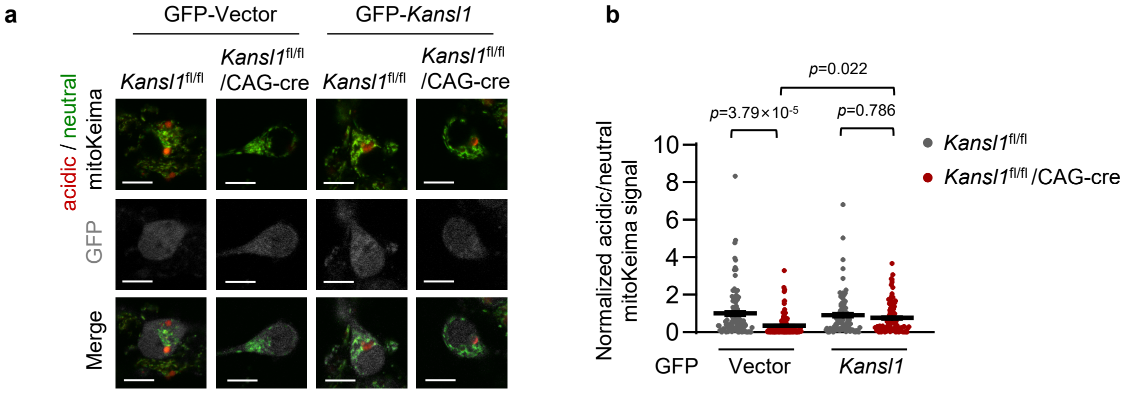

Supplementary Figure 19. Overexpression *Kansl1* can rescue the mitophagic defects in Kansl1-deficient neurons. a, mitoKeima imaging in primary neurons treated with tamoxifen (1 μM, 5 days). Cells were infected with lentivirus expressing T2A-GFP-tagged plasmids. Gray signals in the whole cell correspond to cleaved GFP. The neutral mitoKeima signal is excited at 458 nm (green) and the acid mitoKeima signal is excited at 561 nm (red). Scale bar, 10 μm. **b**, Quantification of mitoKeima signal of cells in (**a**). The acidic/neutral mitoKeima signal in control cells was normalized to ‘1'. n=99,97.85,94 from left to right. Source data are provided as a Source Data file. All data are means ± SEM. All by one-way ANOVA with Tukey's multiple hoc test.

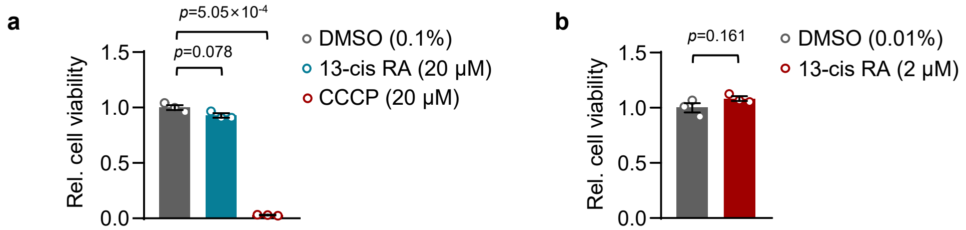

Supplementary Figure 20. 13-cis retinoic acid does not affect cell viability of neurons. a, Primary neurons were treated with 13-cis retinoic acid (20 μM, 24 h) or CCCP(20 μM, 24 h) and cell viability was measured by CellTiter-Glo. n=3 technical replicates. The experiment has been replicated for three time with a similar result. **b,** Primary neurons were treated with 13-cis RA (2 μM, 7 days) and cell viability was measured by CellTiter-Glo. n=3 technical replicates. The experiment has been replicated for three time with a similar result. Cell viability in control cells was normalized to '1'. Source data are provided as a Source Data file. All data are means ± SEM. All by two-tailed Student's *t*-tests.

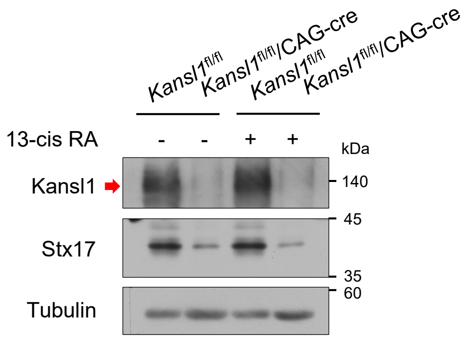

Supplementary Figure 21. 13-cis RA does not affect Kansl1 and Stx17 protein levels. Western blot analysis of primary neurons treated with or without 13-cis retinoic acid (20 μM, 24 h) with the indicated antibodies. The arrow indicated the Kansl1 protein band. Source data are provided as a Source Data file.

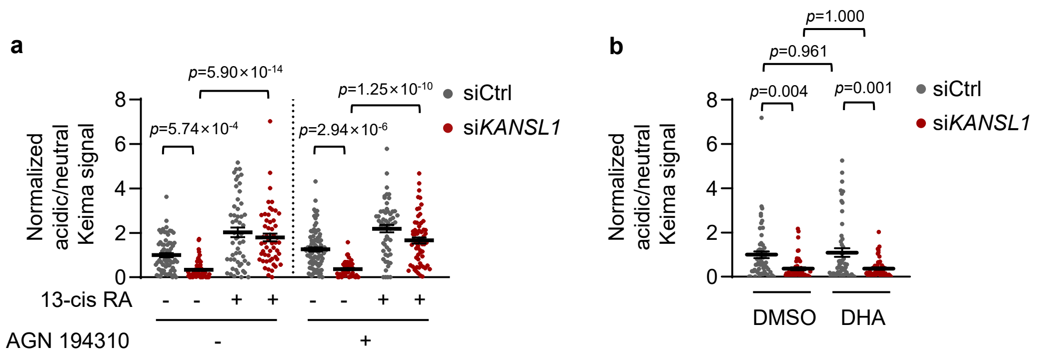

Supplementary Figure 22. 13-cis RA is not likely to regulate autophagy through RAR or RXR. a, Quantification of Keima signal in HeLa cells transfected with indicated siRNAs and cultured in EBSS for 2 h. Cells were pretreated with AGN 194310 (6 nM) for 2 h. Before collected by confocal, cells were cultured with or without 13-cis RA (5 μM) for 24 h. Cell number was obtained from microscopy calculated for 53 to 88 from independent experiments. The acidic/neutral Keima signal in control cells was normalized to '1'. . One-way ANOVA with Sidak's multiple hoc test was used for statistical analysis. **b**, Quantification of Keima signal in HeLa cells transfected with indicated siRNAs and cultured in EBSS for 2 h. Cells were cultured with or without DHA (10 μM) for 24 h. Cell number was obtained from microscopy calculated for 53 to 59 from independent experiments. The acidic/neutral Keima signal in control cells was normalized to '1'. Source data are provided as a Source Data file. All data are means ± SEM. One-way ANOVA with Tukey's multiple hoc test was used for statistical analysis.

Accession	Description	Coverage	Unique Peptides	Score Mascot
157388937	syntaxin-17	15.894	3	25.98
4759154	synaptosomal-associated protein 29	9.690	2	26.07

Supplementary Figure 23. Mass spectrometry analysis of Biotin-13-cis retinoic acid binding peptides. STX17 and SNAP29 peptides identified by mass spectrometry from HeLa cell lysis by Biotin pulldown assay treated with Biotin-13-cis retinoic acid. Source data are provided as a Source Data file.

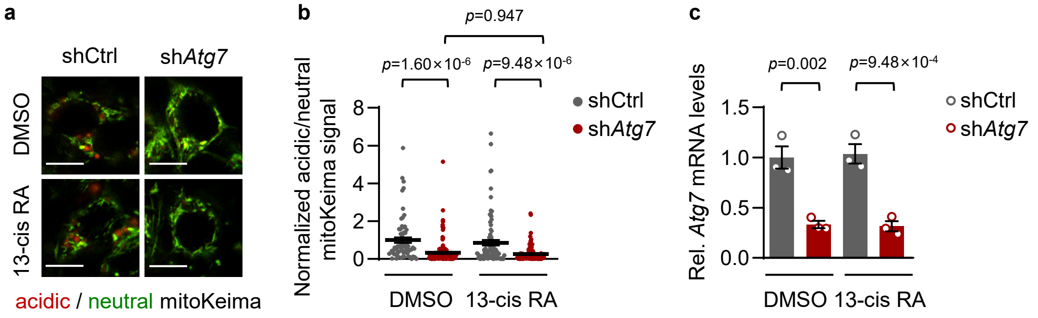

Supplementary Figure 24. 13-cis RA fails to rescue mitophagic defects in *Atg7* knockdown neurons. a, mitoKeima imaging in primary neurons infected with indicated lentivirus expressing shRNAs. Cells were treated with or without 13-cis retinoic acid (20 μM, 24 h). The neutral mitoKeima signal is excited at 458 nm (green) and the acid mitoKeima signal is excited at 561 nm (red). Scale bar, 10 μm. **b**, , Quantification of mitoKeima signal in (**a**). The acidic/neutral mitoKeima signal in control cells was normalized to '1'. n=68,135,87,119 from left to right. **c**, Quantitative real-time PCR analysis of mRNA levels for *Atg7* in (**a**). n=3 technical replicates. Source data are provided as a Source Data file. All data are means ± SEM. All by one-way ANOVA with Tukey's multiple hoc test.

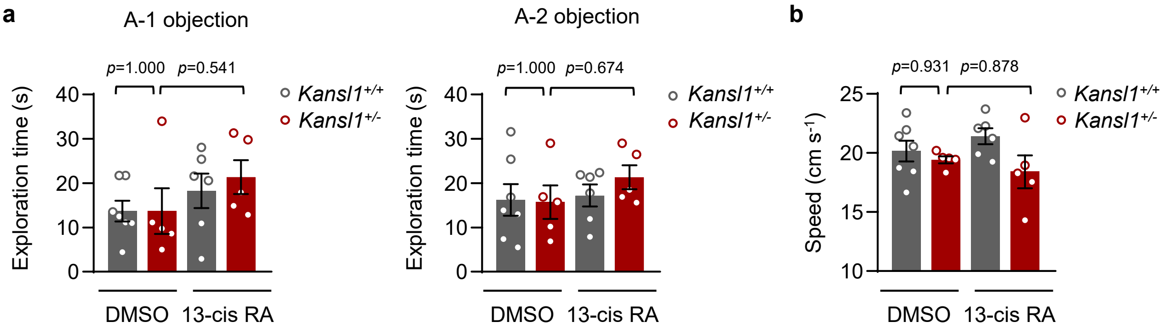

Supplementary Figure 25. Mice administrated with 13-cis RA displayed normal behaviors in tests. a, Object recognition memory of mice was assessed with a retention delay of 24 h. In the first session of test, no difference in object exploration was noticed. n=7,5,6,5 mice from left to right. **b**, The Morris water maze. No difference of swimming speed was observed in different groups. n=7,5,6,5 mice from left to right. Source data are provided as a Source Data file. All data are means ± SEM. All by one-way ANOVA with Tukey's multiple hoc test.

Name	Species	Target sequence (5'-3')
Gapdh-F	Mouse	TGCAGTGGCAAAGTGGAGATT
Gapdh-R	Mouse	GTGAGTGGAGTCATACTGGAACATGT
Kansl1-F	Mouse	GAATATCGAATTCGTCAGCAAACA
Kansl1-R	Mouse	TCTGTTGTATGATCTGGGAAAGG
Pik3r4-F	Mouse	CTGGCGACAGGAGACTGAC
Pik3r4-R	Mouse	GGGGAAACCAAGTCCCAGAA
Atg14-F	Mouse	AGCGGTGATTTCGTCTATTTCG
Atg14-R	Mouse	GCTGTTCAATCCTCATCTTGCAT
Stx17-F	Mouse	TCAAAGTGGCAGGAATTGCAG
Stx17-R	Mouse	AATTTTCCACCTGTGAAGCCTAA
CHIP-*STX17*-P1-F	Human	CACAGGCTTGGTGGGCC
CHIP-*STX17*-P1-R	Human	CTGCTCGCATCGTCTGCG
CHIP-*STX17*-P2-F	Human	CGCAGACGATGCGAGCAG
CHIP-*STX17*-P2-R	Human	GTCACCCACAGCACACC
GAPDH-F	Human	GAGTCAACGGATTTGGTCGT
GAPDH-R	Human	TTGATTTTGGAGGGATCTCG
KANSL1-F	Human	ATCTGATGGCACCTGTGTGG
KANSL1-R	Human	TGAGCTCTCTCCCCTTCTCC
KANSL2-F	Human	TTCGGATTCACGTCTTGCCA
KANSL2-R	Human	AAGCACCTCTGACTGGAACG
KANSL3-F	Human	CTCTCCAGTGTGTCCAGCAG
KANSL3-R	Human	AGCTTCTGGAGCACTTCCTT
WDR5-F	Human	TGTGAAGTTCTCCCCGAACG
WDR5-R	Human	GTGGCCAGTGTACGTCTTCA
MCRS1-F	Human	CGGACTTCGACAACCAGACA
MCRS1-R	Human	TGTGATCTTGGCAGCCTCAG
MOF-F	Human	GTCACGGTGGAGATCGGAGA
MOF-R	Human	CCCTCCTGGTCGTTCACTC
PHF20-F	Human	CAGTGACACCTCCCAGGAAC
PHF20-R	Human	CCTCATCTGGGTTGGTGGTC
PIK3R4-F	Human	CCTGGTCGTTGTGAAGGTTTT
PIK3R4-R	Human	TCTGTGCAGAATTAAGCCTGATT
ATG14-F	Human	GCGCCAAATGCGTTCAGAG
ATG14-R	Human	GCGCCAAATGCGTTCAGAG
STX17-F	Human	GTGAAATTACGCCGTCTTGAAC
STX17-R	Human	GATATTGGATCGGAGTTGCTGAA
NARFL-F	Human	TTAAAAAAATAGGGGTGCACTTCG
NARFL-R	Human	TTCTCGGCATAGCAGATCCAGC
SPICE1-F	Human	GCACAGGGTTTCCAAATGTAAC
SPICE1-R	Human	TCTTCACCATCTACATCATTAAGAGC
NKAP-F	Human	TGAAGATAGCGACAGTGACTCT
NKAP-R	Human	CAGCCTTTGTTCGATCCTTCC
siCtrl	Photinus pyralis	UUCUCCGAACGUGUCACGU
si*KANSL1*-1#	Human	CACUUAGUUCUGAGGUGAA
si*KANSL1*-2#	Human	GUGUCAAAUUGGAGGGUAA
si*NARFL*-1#	Human	GGCCAAGATTCGCATTGAA
si*NARFL*-2#	Human	GACGGGAGCTACTTCCAAA
si*SPICE1*-1#	Human	GTCCCATTGTGGTAAATCA
si*SPICE1*-2#	Human	CAGGAAGAGTTGCCCGTTA
si*NKAP*-1#	Human	GAAGAGTCCGAAGCCCAGCAAATCT
si*NKAP*-2#	Human	GACAAGTGAAGAAATTGCATCATTT
si*MOF*-1#	Human	GGAAUUCUAUGUACACUAC
si*MOF*-2#	Human	GUGAUCCAGUCUCGAGUGA
si*KANSL2*	Human	CGAAUCAGGUUCUCUUCAA
si*KANSL3*	Human	GAGAAGAUUCGAGCUGAGA
si*WDR5*	Human	CCAAUUCUAACACUGUCUA
si*MCRS1*	Human	CGAUUCGUCUUCCUUAUCA
si*PHF20*	Human	CCACUAAUGGGACCCAUGA
sh*KANSL1*-F	Human	CCGGCTCGTAAGGACAGGCACAAATCTCGAGATTTGTGCCTGTC CTTACGAGTTTTTG
sh*KANSL1*-R	Human	AATTCAAAAACTCGTAAGGACAGGCACAAATCTCGAGATTTGTG CCTGTCCTTACGAG
sh*Stx17*-F	Mouse	CCGGTCAGAGTCTGACTCAGATATACTCGAGTATATCTGAGTCA GACTCTGATTTTTG
sh*Stx17*-R	Mouse	AATTCAAAAATCAGAGTCTGACTCAGATATACTCGAGTATATCTG AGTCAGACTCTGA
sh*Atg7*-F	Mouse	CCGGTGAGATCTGGGAAGCCATAAACTCGAGTTTATGGCTTCCC AGATCTCATTTTTG
sh*Atg7*-R	Mouse	AATTCAAAAATGAGATCTGGGAAGCCATAAACTCGAGTTTATGG CTTCCCAGATCTCA

Supplementary Table 1. Nucleotide sequences used in this study.
