# Supplementary material for: Kansl1 haploinsufficiency impairs autophagosome-lysosome fusion and links autophagic dysfunction with Koolen-de Vries syndrome in mice
Source: Nat Commun. 2022 Feb 17;13:931. doi: 10.1038/s41467-022-28613-0 (PMC8854428; doi:10.1038/s41467-022-28613-0)
Supplement: Supplementary file 2 — Description of Additional Supplementary Files [file 41467_2022_28613_MOESM2_ESM.docx]

**Description of Additional Supplementary Files**

**File Name:** Supplementary Data 1

**Description:** Mass spectrometry data for identifying binding targets of biotin-13-cis retinoic acid.
